# Combining SiRP**α** decoy–coengineered T cells and antibodies augments macrophage-mediated phagocytosis of tumor cells

**DOI:** 10.1172/JCI161660

**Published:** 2024-04-23

**Authors:** Evangelos Stefanidis, Aikaterini Semilietof, Julien Pujol, Bili Seijo, Kirsten Scholten, Vincent Zoete, Olivier Michielin, Raphael Sandaltzopoulos, George Coukos, Melita Irving

**Affiliations:** 1 Ludwig Institute for Cancer Research, Department of Oncology, University of Lausanne (UNIL) and University Hospital of Lausanne (CHUV), Lausanne, Switzerland.; 2 Department of Molecular Biology and Genetics, Democritus University of Thrace, Alexandroupolis, Greece.; 3 Swiss Institute of Bioinformatics, Lausanne, Switzerland.; 4 Precision Oncology, University Hospital of Geneva (HUG), Geneva, Switzerland.

**Keywords:** Immunology, Therapeutics, Cancer immunotherapy, Innate immunity, T cells

## Abstract

The adoptive transfer of T cell receptor–engineered (TCR-engineered) T cells (ACT) targeting the HLA-A2–restricted cancer-testis epitope NY-ESO-1_157–165_ (A2/NY) has yielded favorable clinical responses against several cancers. Two approaches to improve ACT are TCR affinity optimization and T cell coengineering to express immunomodulatory molecules that can exploit endogenous immunity. By computational design we previously developed a panel of binding-enhanced A2/NY-TCRs including A97L, which augmented the in vitro function of gene-modified T cells as compared with WT. Here, we demonstrated higher persistence and improved tumor control by A97L–T cells. In order to harness macrophages in tumors, we further coengineered A97L–T cells to secrete a high-affinity signal regulatory protein α (SiRPα) decoy (CV1) that blocks CD47. While CV1-Fc–coengineered A97L–T cells mediated significantly better control of tumor outgrowth and survival in Winn assays, in subcutaneous xenograft models the T cells, coated by CV1-Fc, were depleted. Importantly, there was no phagocytosis of CV1 monomer–coengineered T cells by human macrophages. Moreover, avelumab and cetuximab enhanced macrophage-mediated phagocytosis of tumor cells in vitro in the presence of CV1 and improved tumor control upon coadministration with A97L–T cells. Taken together, our study indicates important clinical promise for harnessing macrophages by combining CV1-coengineered TCR–T cells with targeted antibodies to direct phagocytosis against tumor cells.

## Abstract 2

The combination of tumor-specific antibodies and decoys of the innate immune checkpoint CD47 secreted by gene-modified T cells potentiate targeted phagocytosis of tumor cells.

## Graphical abstract

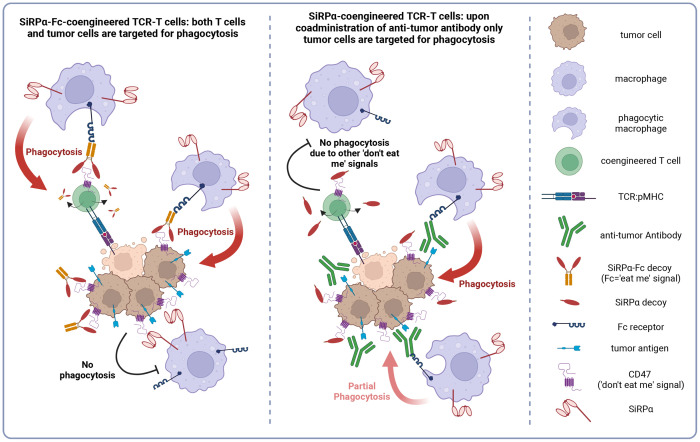

## Introduction

The cancer-testis antigen NY-ESO-1 is a favorable target for immunotherapy development because it is expressed by a broad range of cancers but in healthy adult tissues it is restricted to male germ cells (1–3). Since NY-ESO-1 is a self-antigen, T cell receptors (TCRs) recognizing A2/NY tend to bind weakly, but higher-affinity variants of the well-characterized A2/NY-TCR 1G4 (4) have demonstrated promising ACT responses in the clinic against melanoma, synovial cell carcinoma, and myeloma (5–8). To improve ACT it is also critical to address the tumor microenvironment (TME). Indeed, tumors can be highly immunosuppressive, but coadministration of radiotherapy (9), chemotherapy, or immune checkpoint inhibitors (10), for example, can be used to reprogram the TME to better support the transferred T cells and harness endogenous immunity (11, 12). Alternatively, T cells can be rationally coengineered to express immunomodulatory molecules directly in tumors (13, 14).

CD47, a “don’t eat me” signal recognized by signal regulatory protein α (SiRPα) on phagocytes, is a cell membrane glycoprotein expressed by most healthy cells that plays an important role in cell clearance and tissue homeostasis (15). Numerous studies have reported the upregulation of CD47 as an innate immune evasion strategy by both liquid and solid cancers (16–18). In xenograft models (17, 19–22), CD47/SiRPα axis blockade has been associated with enhanced phagocytic activity of macrophages, while in syngeneic models (23–26), dendritic cells have emerged as key players (26).

However promising, CD47 blockade is hampered by the large CD47 antigen sink, treatment-related toxicities, and lack of therapeutic efficacy (27, 28). To address these hurdles, we have taken a T cell coengineering approach to limit blockade of the CD47/SiRPα axis to within the TME. In our study, we coengineered human T cells to express an affinity-optimized A2/NY-TCR (29–31) and to secrete a previously described binding-enhanced variant of the SiRPα ectodomain, CV1 (28), either as an Fc fusion protein or as a monomer. Human T cells gene-engineered to secrete CV1-Fc or CV1 (constitutively or inducibly) became coated in the decoys and were depleted in vivo in NOD *scid* gamma (NSG) mice. However, human T cells secreting CV1 (monomer) were not targeted by human macrophages in vitro, and CV1 in combination with cetuximab and/or avelumab augmented phagocytosis of tumor cells. Taken together, our data indicate important clinical promise for this combinatorial ACT strategy, which harnesses both adaptive and innate immunity while avoiding T cell depletion by phagocytes and lowering the risk of toxicity in patients.

## Results

### Superior tumor control by T cells expressing an affinity-optimized A2/NY-TCR.

**Figure 1:**
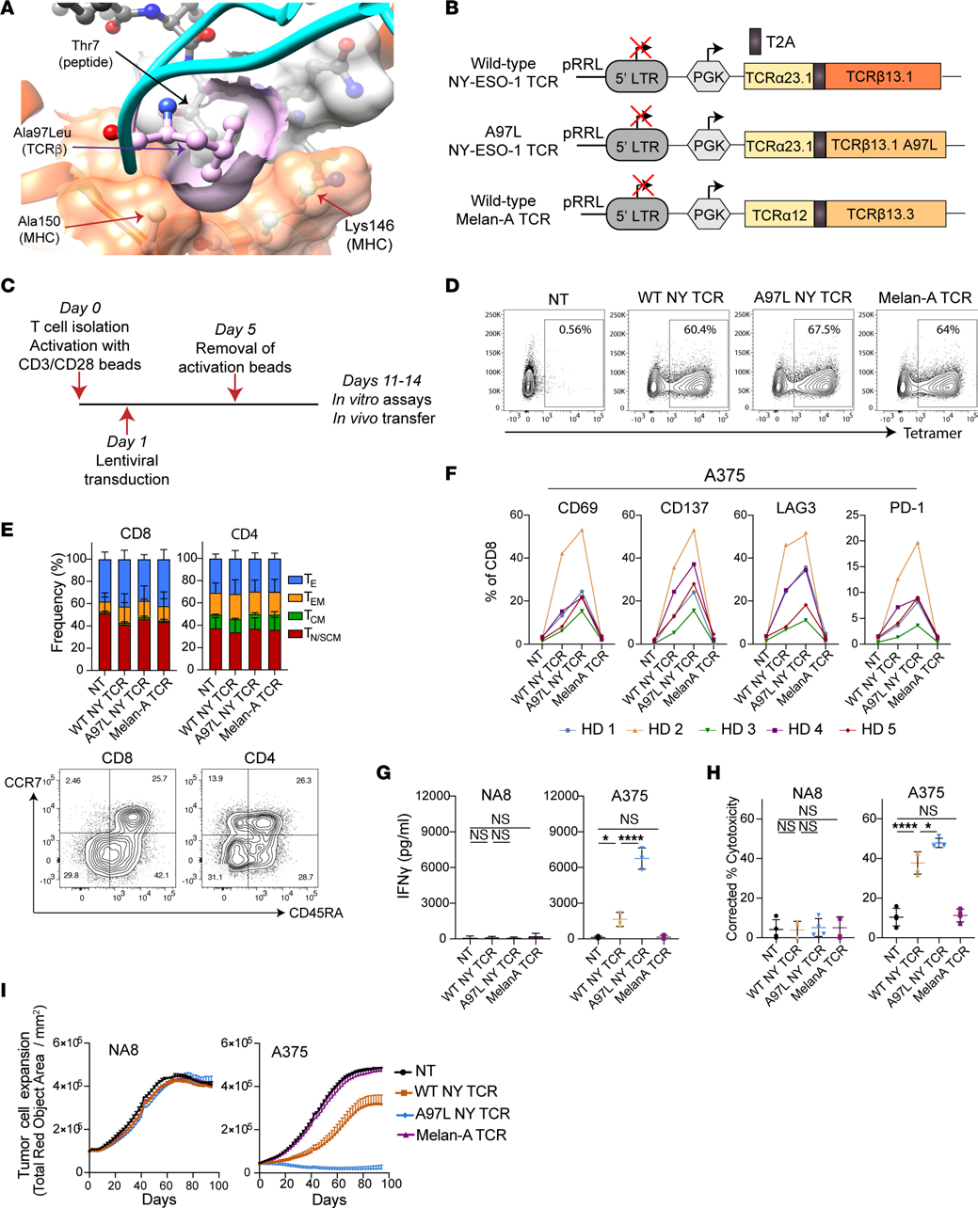
T cells expressing affinity-enhanced A2/NY-TCR A97L exhibit superior effector function in vitro compared with WT. (**A**) TCR variant A97L binding to A2/NY-ESO-1_157–165_ (Protein Data Bank ID 2BNR). A97L replacement in CDR3β (in ball and stick representation, colored in pink) enhances direct peptide contact via non-polar interactions with Thr7 (ball and stick representation, in gray, below TCRβ chain) as well as with MHC-Ala150 and -Lys146 (ball and stick representation, surface in orange). (**B**) Schematic of lentiviral constructs encoding TCRs. (**C**) Strategy for T cell activation, transduction, and expansion. (**D**) Transduction efficiency of CD8^+^ T cells with TCRs evaluated by tetramer staining (data are representative of 5 donors). (**E**) Top: Frequency of effector and memory phenotypes of rested CD8^+^ and CD4^+^ T cells transduced to express the different TCRs (*n* = 3). Bottom: Representative flow cytometric analysis of anti-CCR7 and anti-CD45RA Ab–stained T cells (T_E_, effector; T_EM_, effector memory; T_CM_, central memory; T_N/SCM_, naive/stem cell–like memory). (**F**) Expression (frequency) of activation markers and checkpoint receptors on CD8^+^ TCR-engineered T cells 24 hours after stimulation with A375 tumor cells (*n* = 5). HD, healthy donor. (**G**) IFN-γ secretion levels by TCR-modified T cells 24 hours after stimulation with NA8 and A375 tumor cells at effector/target ratio (E/T) = 1:1 (*n* = 3). (**H**) Frequency of annexin V^+^ DAPI^+^ cells, corrected to tumor alone, in 24-hour cocultures of NA8 and A375 tumor cells with TCR-modified T cells at E/T = 1:1 (*n* = 4). (**I**) Evaluation of mKate2^+^ NA8 and A375 tumor cell growth control over time by TCR–T cells at E/T = 1:1 using live-cell IncuCyte imaging (data are representative of 4 donors). Statistical analysis was done by 1-way ANOVA (**E**, **G**, and **H**) with correction for multiple comparisons by post hoc Tukey’s test (**E**, **G**, and **H**). *P < 0.05; ****P< 0.0001.

Previously, by structure-based computational design (29), we developed a panel of increasing-affinity A2/NY-TCRs and demonstrated maximum effector function for T cells expressing TCR variants in the upper range of natural affinity (30, 31). One of the TCRs comprising a single amino acid replacement in the β chain (A97L; Figure 1A) was rationally designed to increase NY-peptide contact (30, 31). Here, we began by subcloning both the A2/NY WT-TCR and the A97L-TCR into lentiviral transfer vectors, as well as an HLA-A2/Melan-A_26–35_–directed TCR as a negative control (Figure 1B) (32). Primary human T cells were subsequently transduced and expanded (Figure 1C), and tetramer staining revealed similar high cell-surface expression levels of the TCRs on CD8^+^ T cells (Figure 1D). Because the WT-TCR is CD8 dependent (30), anti-Vβ13.1 antibody (Ab) was used to detect its expression by CD4^+^ T cells (Supplemental Figure 1A; supplemental material available online with this article; https://doi.org/10.1172/JCI161660DS1). We observed similar expansion (Supplemental Figure 1B) as well as proportion of effector, effector memory, central memory, and naive/stem cell–like phenotypes for the different TCR–T cells (Figure 1E and Supplemental Figure 1C).

Coculture with the A2^+^/NY^+^ melanoma cell line A375 revealed consistently higher upregulation of the activation markers CD69 and CD137 as well as of the inhibitory markers lymphocyte-activation gene 3 (LAG3) and programmed cell death protein 1 (PD-1) for CD8^+^ A97L–T cells as compared with WT (Figure 1F and Supplemental Figure 1D). We also observed significantly higher IFN-γ production and cytotoxicity by A97L–T cells than WT (Figure 1, G and H). Similar results were acquired for cocultures with the A2^+^/NY^+^ osteosarcoma cell line Saos-2 (Supplemental Figure 1, E and F). Likewise, in an IncuCyte real-time cytotoxicity assay, A97L–T cells were far more potent than WT against A375 (Figure 1I). There was no reactivity of the WT– and A97L–T cells against the A2^+^/NY^–^ melanoma tumor cell line NA8, nor did Melan-A–TCR–T cells show any reactivity above background levels against NA8, A375, and Saos-2 (Figure 1, F–I, and Supplemental Figure 1, D–G).

**Figure 2:**
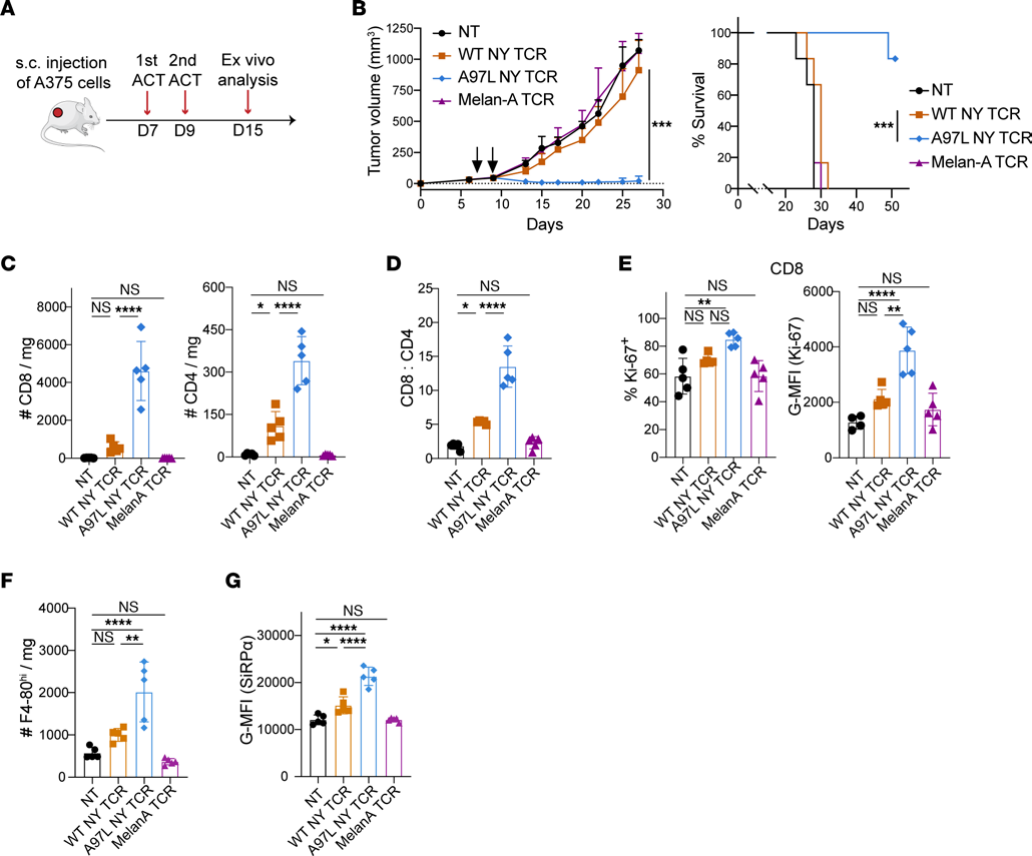
Affinity-enhanced A97L-TCR-T cells significantly improve tumor control and survival. (**A**) Schematic of ACT study. (**B**) Control of A375 tumors (left) in NSG mice and survival curves (right) following ACT (*n* = 6 mice per group; data are representative of 2 independent studies). (**C**) Number of intratumoral human CD8^+^ (left) and CD4^+^ (right) T cells per milligram of tumor 7 days after ACT (*n* = 5; data are representative of 2 independent studies). (**D**) Ratio of intratumoral CD8^+^/CD4^+^ human T cell frequency 7 days after ACT (*n* = 5; data are representative of 2 independent studies). (**E**) Frequency (left) and geometric mean fluorescence intensity (G-MFI) (right) of Ki-67 expression within intratumoral human CD8^+^ T cells 7 days after ACT (*n* ≥4; data are representative of 2 independent studies). (**F**) Number of intratumoral mouse F4-80^hi^ macrophages per milligram of tumor 7 days after ACT (*n* = 5; data are representative of 2 independent studies). (**G**) G-MFI of mouse SiRPα expression within intratumoral mouse F4-80^hi^ macrophages 7 days after ACT (*n* = 5; data are representative of 2 independent studies). Statistical analysis was done by 2-way ANOVA (**B**, left), Mantel-Cox (**B**, right), or 1-way ANOVA (**C**–**G**) with correction for multiple comparisons by post hoc Tukey’s test (**B**–**G**). *P < 0.05; **P < 0.01; ***P < 0.001; ****P< 0.0001.

We subsequently performed ACT studies in NSG mice bearing subcutaneous A375 tumors (Figure 2A) and observed significantly improved tumor control and survival (Figure 2B) upon transfer of A97L T cells. Ex vivo characterization at day 7 after ACT revealed significantly higher intratumoral presence of both CD8^+^ and CD4^+^ A97L–T cells than WT (Figure 2C), as well as a significantly higher CD8/CD4 ratio for A97L–T cells (Figure 2D). In addition, the infiltrating CD8^+^ A97L–T cells were characterized by higher Ki-67 levels (Figure 2E), indicative of superior proliferative capacity. Despite the elevated numbers of CD4^+^ A97L–T cells in tumors (Figure 2C), there were no differences in Ki-67 expression (Supplemental Figure 1H), suggesting superior homing and/or persistence. Notably, ACT with A97L–T cells was accompanied by a significantly increased presence of intratumoral F4-80^hi^ macrophages (Figure 2F) expressing higher levels of SiRPα (Figure 2G), which may counter tumor control.

### CV1-Fc promotes macrophage-mediated phagocytosis of tumor cells.

The previously described SiRPα ectodomain variant CV1 is of high affinity for CD47, small in size, and has demonstrated low toxicity upon systemic administration as compared with Ab-based inhibitors (28). Based on these favorable properties, we next set out to coengineer A97L–T cells to secrete CV1. We began by validating the binding and functionality of soluble recombinant proteins comprising the CD47-binding ectodomain of human SiRPα (amino acids 27–371) fused to human IgG1-Fc, which has been shown to enhance tumor cell phagocytosis by macrophages (33). Throughout our study, we compared CV1 with WT as well as an inactive (in) (34) variant of the SiRPα ectodomain (Supplemental Figure 2A). The A2^+^/NY^+^ tumor cell lines Me275, A375, and Saos-2 express CD47 (Supplemental Figure 2B, top), and we observed higher binding (Supplemental Figure 2B, bottom) as well as saturation of binding at lower concentrations (Supplemental Figure 2C) for CV1-SiRPα-Fc (CV1-Fc) than wtSiRPα-Fc recombinant fusion proteins. There was no CD47 binding by inSiRPα-Fc (Supplemental Figure 2B, bottom, and Supplemental Figure 2C), nor was there binding by any of the fusion proteins to CD47-deficient JinB8 Jurkat cells (Supplemental Figure 2D).

To compare the ability of the SiRPα-Fc molecules to promote phagocytosis, we set up cocultures of human CD14^+^ monocyte-derived macrophages (MDMs) with PKH26 fluorescently prelabeled tumor cells; the cells were analyzed after 4 hours by flow cytometry (gating strategy shown in Supplemental Figure 2E). CV1-Fc promoted a significantly higher level of tumor cell phagocytosis by MDMs than wtSiRPα-Fc, and the inactive variant had no biological impact (Supplemental Figure 2F). By Amnis ImageStream imaging we were able to visualize phagocytosis (Supplemental Figure 2G).

**Figure 3:**
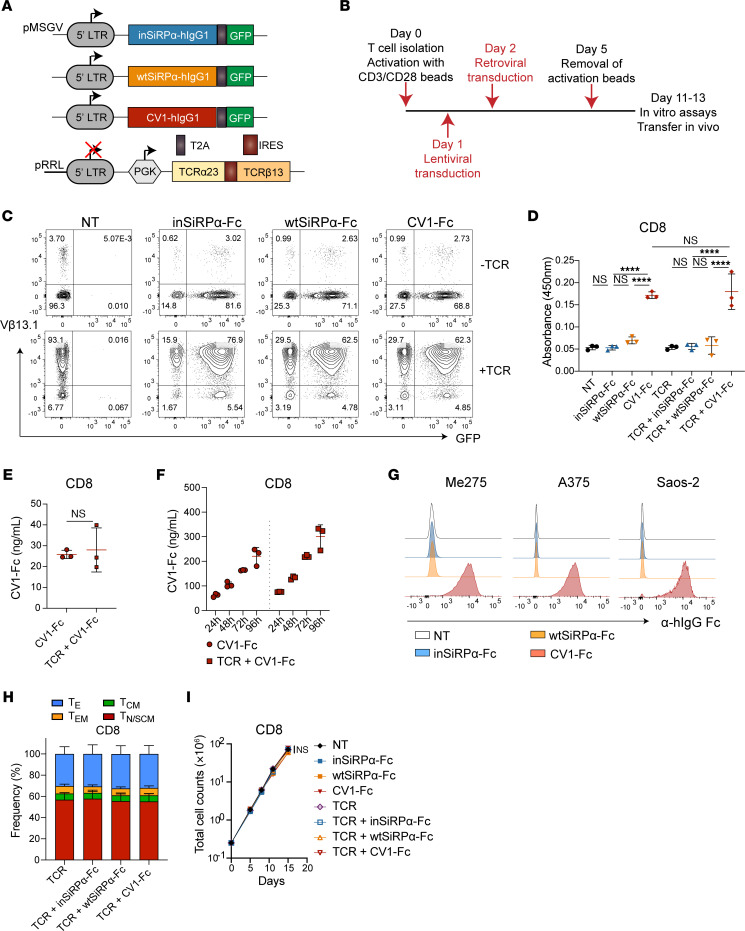
SiRPα-Fc decoys are efficiently produced by gene-modified T cells. (**A**) Schematic of retroviral constructs encoding inSiRPα-Fc, wtSiRPα-Fc, or CV1-Fc proteins with an EGFP reporter gene, and of a lentiviral construct encoding the A97L-TCR. (**B**) Strategy for T cell activation, dual virus transduction, and expansion. (**C**) Expression of SiRPα-Fc and A97L-TCR in cotransduced rested human CD8^+^ T cells as detected by EGFP and anti–human Vβ13.1 Ab, respectively (data are representative of 12 independent donors). (**D**) CD47-based ELISA detection of SiRPα-Fc secreted by engineered CD8^+^ T cells (*n* = 3). (**E**) Quantification of CD8^+^ T cell–secreted CV1-Fc by CD47-based ELISA after 24 hours of culture (*n* = 3). (**F**) Quantification of CV1-Fc accumulated in culture supernatants of engineered CD8^+^ T cells over time (representative results for 3 donors). (**G**) Binding of CD8^+^ T cell–secreted CV1-Fc on different CD47^+^ tumor cell lines (data are representative of 3 donors). (**H**) Frequency of effector and memory phenotypes of transduced and rested CD8^+^ T cells (*n* = 3) (T_E_, effector; T_EM_, effector memory; T_CM_, central memory; T_N/SCM_, naive/stem cell–like memory). (**I**) Expansion of engineered CD8^+^ T cells (*n* = 3). Statistical analysis was done by 1-way ANOVA (**D** and **H**), unpaired 2-tailed *t* test (**E**), or 2-way ANOVA (**I**) with correction for multiple comparisons by post hoc Tukey’s test on pooled donors (**D**, **H**, and **I**). ****P< 0.0001.

We next built retroviral cassettes for CV1-Fc, wt-Fc, and inSiRPα-Fc, each along with GFP as a marker of transduction (Figure 3A), and then cotransduced T cells with lentivirus encoding TCR and, 24 hours later, with retrovirus to coexpress the decoys (Figure 3B). The use of lenti- and retroviruses packaged with VSV-G and RD114 (35), respectively, was undertaken to abrogate virus envelope glycoprotein competition for target receptors enabling T cell infection. Our mixed virus strategy yielded high dual transduction efficiency in both CD8^+^ (>60%) and CD4^+^ (>50%) T cells (Figure 3C and Supplemental Figure 3A).

A CD47-based ELISA confirmed T cell secretion of the decoys (Figure 3D and Supplemental Figure 3B) and that similar quantities of CV1-Fc were produced upon cotransduction to express the A97L-TCR (Figure 3E and Supplemental Figure 3C). CV1-Fc accumulated in the culture supernatant of the gene-modified T cells over time, reaching approximately 300 ng/mL for CD8^+^ A97L–T cells (Figure 3F) and 450 ng/mL for CD4^+^ A97L–T cells (Supplemental Figure 3D) at 96 hours. Notably, this is almost an order of magnitude lower than the concentration typically used for CD47 blocking Abs and recombinant SiRPα-Fc proteins (28) and likely explains the detection of T cell–secreted CV1-Fc but not wtSiRPα-Fc binding to CD47^+^ tumor cells (Figure 3G and Supplemental Figure 3E) from culture supernatants. We observed no differences in memory phenotype (Figure 3H and Supplemental Figure 3F) nor expansion (Figure 3I and Supplemental Figure 3G) between the differently engineered T cells.

### CV1-Fc secreted by TCR–T cells augments phagocytosis by macrophages.

We next sought to evaluate the activity of SiRPα-Fc decoys secreted by gene-modified T cells. First, however, we tested whether the decoys had any direct impact on T cell function in the presence of target tumor cells (Supplemental Figure 4A). While there were no differences in target cell killing by TCR–T cells with versus without decoy (Supplemental Figure 4, B–D), we consistently observed higher IFN-γ production by CV1-Fc–coengineered TCR–T cells (Supplemental Figure 4E), in line with previous work demonstrating a costimulatory role for CD47 on T cells (36).

**Figure 4:**
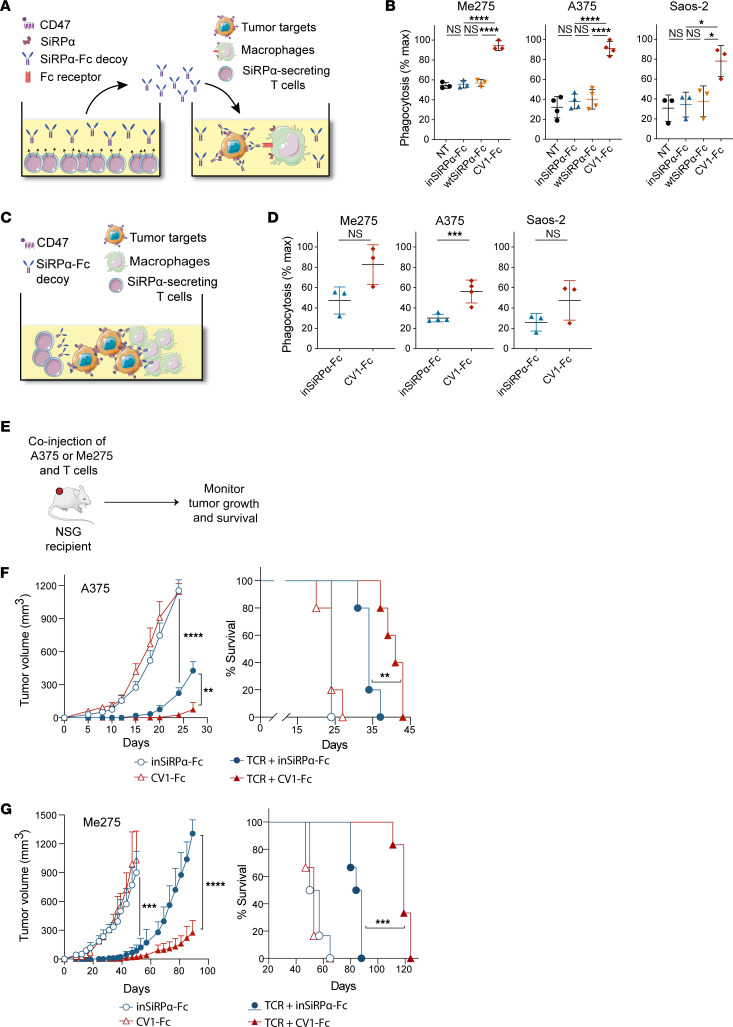
CV1-Fc produced by TCR-T cells augments tumor cell phagocytosis by macrophages and increases control of tumor outgrowth. (**A**) Schematic of tumor cell phagocytosis by macrophages (human MDMs) in the presence of supernatant from SiRPα-Fc–engineered T cells. (**B**) Tumor cell phagocytosis by MDMs in the presence of supernatant from SiRPα-Fc–engineered T cells (*n* ≥3). (**C**) Schematic of triple coculture of tumor cells, macrophages (MDMs), and engineered T cells. (**D**) Tumor cell phagocytosis by MDMs in triple cocultures with SiRPα-Fc–secreting T cells (*n* ≥3). (**E**) Schematic of the Winn assay. (**F**) Control of A375 outgrowth (left) and survival (right) in a Winn assay with SiRPα decoy–coengineered A97L–T cells (*n* = 5; data are representative of 2 independent studies). (**G**) Control of Me275 outgrowth (left) and survival (right) in a Winn assay with SiRPα decoy–coengineered A97L–T cells (*n* = 6; data are representative of 2 independent studies). Statistical analysis was done by 1-way ANOVA (**B** and **D**), 2-way ANOVA (**F**, left, and **G**, left), or Mantel-Cox (**F**, right, and **G**, right) with correction for multiple comparisons by post hoc Tukey’s test (**B** and **D**; and **F** and **G**: CV1-Fc versus TCR + inSiRPα-Fc) or post hoc Šidák’s test (**F** and **G**: TCR + inSiRPα-Fc vs. TCR + CV1-SiRPα-Fc). *P < 0.05; **P < 0.01; ***P < 0.001; ****P< 0.0001.

Cocultures of human CD14^+^ MDMs with tumor cells in the presence of supernatants collected from decoy-engineered T cell cultures (Figure 4A) revealed significantly higher phagocytic activity for CV1-Fc than wtSiRPα-Fc (Figure 4B), and supernatants from CV1-Fc–engineered T cells had a similar impact on phagocytosis regardless of whether or not the T cells were coengineered to express the A97L-TCR (Supplemental Figure 4F). We then set up triple coculture assays comprising target tumor cells, MDMs, and the differently gene-modified T cells (schematic in Figure 4C) and consistently observed (even in just a 4-hour period) higher phagocytic activity in the presence of CV1-Fc–engineered T cells than ones expressing inSiRPα-Fc (Figure 4D).

Finally, a side-by-side comparison of the 3 coculture conditions — (a) tumor cells and MDMs plus recombinant CV1-Fc; (b) tumor cells, MDMs, and CV-Fc–engineered T cells; and (c) tumor cells and MDMs plus CV1-Fc supernatant (Supplemental Figure 4G) — revealed that in some instances the extent of phagocytosis achieved was superior with CV1-Fc culture supernatants as compared with the addition of saturating amounts of recombinant CV1-Fc (10 μg/mL; Supplemental Figure 4H).

### Enforced expression of CV1-Fc by TCR–T cells improves the control of tumor outgrowth, but in a subcutaneous ACT model the T cells are depleted.

Next, we performed an in vivo Winn assay in which NSG mice were subcutaneously coinjected with tumor cells plus the gene-modified T cells and control of tumor outgrowth was evaluated over days (Figure 4E). We chose NSG mice as a model based on the high-affinity cross-species reactivity of NSG-SiRPα with human CD47 (37). We observed significantly improved control of tumor outgrowth and survival in the context of both A375 (Figure 4F) and Me275 (Figure 4G) for CV1-Fc– as compared with inSiRPα-Fc–coengineered A97L–T cells. A complete lack of tumor control and poor survival were observed upon treatment with T cells engineered to express decoys but not the TCR (Figure 4, F and G).

**Figure 5:**
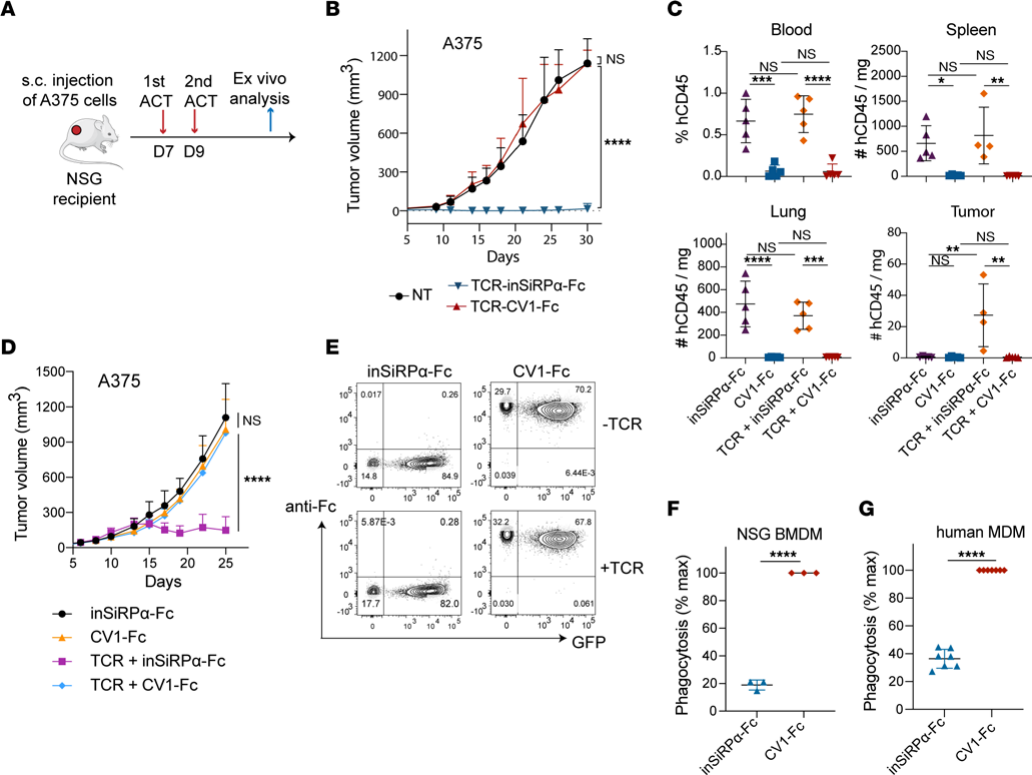
CV1-Fc-engineered T cells are phagocytosed by macrophages in vitro and depleted in vivo. (**A**) Schematic of ACT against subcutaneous A375 tumors and ex vivo analysis. (**B**) A375 tumor growth and control curves following ACT (*n* = 7). (**C**) Frequency and number of human CD45^+^ cells in harvested tissues 5 days after ACT (*n* ≥4; data are representative of 2 independent studies). (**D**) A375 tumor growth and control curves following ACT supplemented with coadministration of soluble inSiRPα-Fc and CV1-Fc proteins (*n* ≥5). (**E**) Flow cytometry detection of T cell–secreted CV1-Fc binding on T cell surface CD47 by anti–human IgG-Fc Ab staining (data are representative of 6 donors). (**F**) Phagocytosis of T cells coated with secreted CV1-Fc by NSG BMDMs in vitro (*n* = 3). (**G**) Phagocytosis of T cells coated with secreted CV1-Fc by MDMs in vitro (*n* = 7). Statistical analysis was done by 2-way ANOVA (**B** and **D**), 1-way ANOVA (**C**), or unpaired 2-tailed *t* test (**F** and **G**) with correction for multiple comparisons by post hoc Tukey’s test (**B**–**D**). *P < 0.05; **P < 0.01; ***P < 0.001; ****P< 0.0001.

In a subcutaneous tumor model (Figure 5A) we unexpectedly observed that tumor control was abrogated for TCR–T cells expressing the high-affinity decoy but not the inactive one (Figure 5B). Inspection of the blood, spleen, lung, and tumors after ACT revealed depletion of CV1-Fc–coengineered T cells (with or without TCR) (Figure 5C). We further observed that coadministration of recombinant CV1-Fc with A97L–T cells resulted in complete tumor escape (Figure 5D). We reasoned that this may be due to binding of the high-affinity decoy to CD47 on the T cell surface and subsequent macrophage-mediated depletion. Indeed, by flow cytometric analysis we observed T cell coating by CV1-Fc (presumably occurring in both an autocrine and a paracrine manner within the culture medium) but not by inSiRPα-Fc (Figure 5E), and coculture assays revealed phagocytosis by both NSG bone marrow–derived macrophages (BMDMs) and human MDMs of T cells expressing CV1-Fc but not inSiRPα-Fc (Figure 5, F and G). Morphology, shape, and, in particular, the relatively small size of the T cells (as compared with tumor cells) may contribute to their high susceptibility to phagocytosis once CD47 is blocked by CV1-Fc.

### Activation-inducible expression of CV1-Fc or use of wtSiRPα-Fc or a weaker Fc tail cannot prevent T cell depletion.

In an effort to rescue CV1-Fc–coengineered T cells from depletion in vivo, we next generated lentiviral vectors encoding the decoys under the activation-inducible promoter nuclear factor of activated T cells (NFAT_6_) response elements fused to the IL-2 minimal promoter (6xNFAT; Supplemental Figure 5A) (14). Such inducible expression of gene cargo has been used to alleviate systemic toxicity (38–40), and we hypothesized that restricting decoy production to within the TME would favor tumor cell binding. We also optimized a cotransduction protocol so that the T cells were not coated in decoy during manufacturing (Supplemental Figure 5, B–F). While activation-inducible expression of CV1-Fc transiently improved control, the T cells were depleted and the tumors mostly escaped (Supplemental Figure 5, G and H).

We further questioned whether substitution IgG1-Fc (33) with IgG4-Fc (which binds Fc receptors [FcRs] more weakly; ref. 41) fused to CV1 could rescue the coengineered T cells from depletion, but this was not the case (Supplemental Figure 5I). Moreover, TCR–T cells coengineered to express wtSiRPα-Fc decoy were depleted in vivo (Supplemental Figure 5I) even though we could not detect its binding to tumor cells in vitro (Figure 3G). We hypothesized that there might be an avidity effect in vivo, supported by the observation that immobilization of wtSiRPα-Fc on protein G–coated beads enabled tumor cell binding levels similar to those of the high-affinity decoy (Supplemental Figure 5J).

### Tumor-specific Abs combined with CV1 potentiate macrophage-mediated phagocytosis of tumor cells.

Previous studies have demonstrated that CD47 blockade with monomeric SiRPα decoys can lower the phagocytic threshold, but macrophage mobilization and target cell engulfment further require prophagocytic signals such as via Fc/FcR engagement (23, 28). We thus reasoned that we could remove the prophagocytic IgG1-Fc tail from the CV1 decoy to spare the T cells from depletion and instead coadminister tumor-targeting Ab(s) comprising an active Fc tail (Supplemental Figure 6A).

We identified epidermal growth factor receptor (EGFR), human epidermal growth factor receptor 2 (HER2), melanoma-associated chondroitin sulfate proteoglycan (MCSP), and programmed death ligand 1 (PD-L1) as being expressed by A375 tumor cells (Supplemental Figure 6B, top panel) and by established tumors (Supplemental Figure 6B, bottom panel). EGFR, HER2, and MCSP are all cell-surface receptors commonly deregulated in cancer, and PD-L1 is an immune checkpoint receptor (42). We acquired the clinically approved monoclonal Abs cetuximab, trastuzumab, and avelumab, targeting EGFR, HER2, and PD-L1, respectively, which each comprise a functional IgG1-Fc tail needed for Ab-dependent cellular phagocytosis (ADCP) by macrophages. To target MCSP, an in-house human IgG1-Fc fusion Ab was generated using publicly available scFv sequences. Aside from transient upregulation of PD-L1 during activation (43), none of the antigens were present on the surface of cultured T cells (Supplemental Figure 7, A and B).

**Figure 6:**
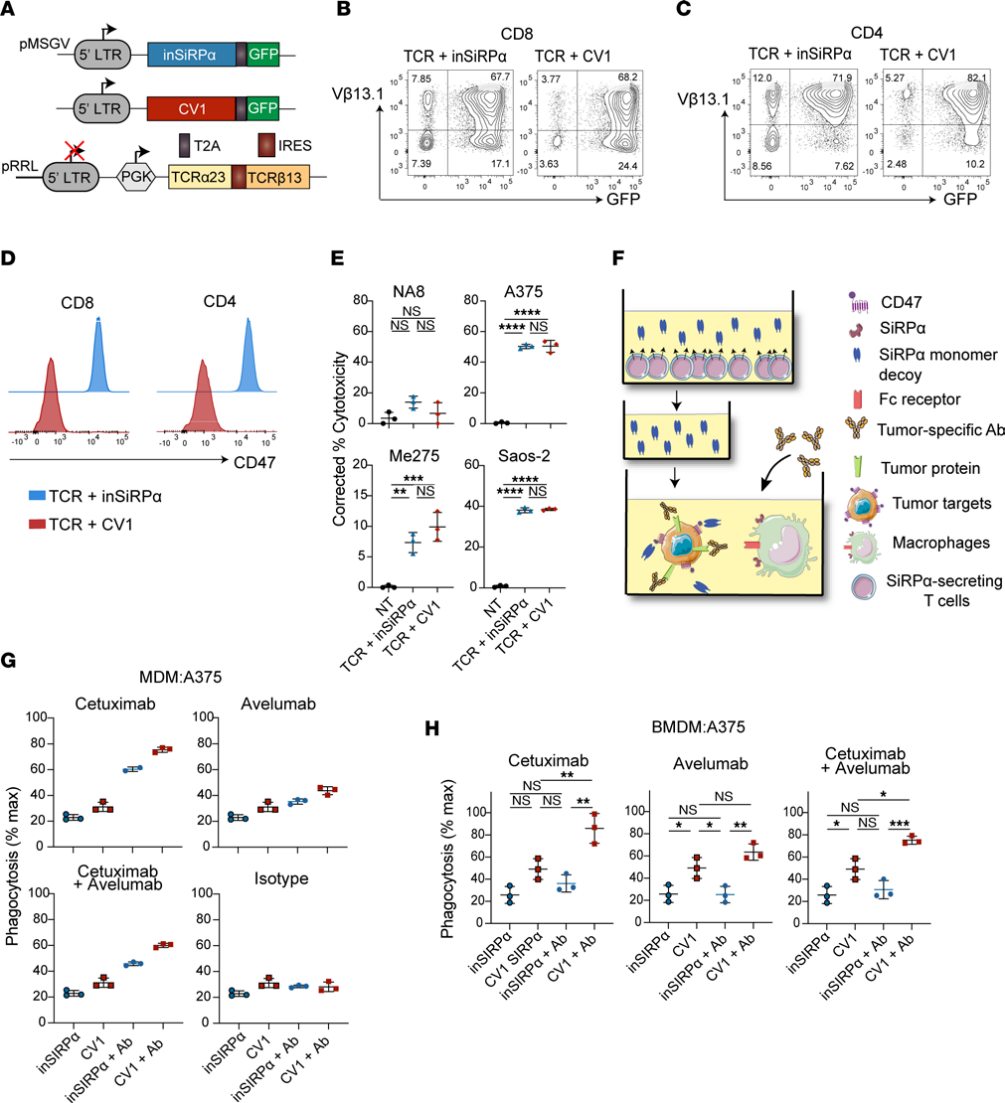
Tumor-targeted monoclonal Abs synergize with T cell-secreted CV1 monomer to augment tumor cell phagocytosis by macrophages in vitro. (**A**) Schematic of retroviral vectors encoding SiRPα monomers and of lentiviral vector encoding A97L-TCR. (**B** and **C**) Expression of SiRPα monomer and A97L-TCR in transduced CD8^+^ (**B**) and CD4^+^ (**C**) T cells, detected by EGFP and anti-Vβ13.1 Ab staining, respectively (data are representative of 11 donors). (**D**) Flow cytometric detection of CV1 monomer binding on CD8^+^ and CD4^+^ T cells by comparison of anti-CD47 Ab staining to CV1- versus inSiRPα-engineered T cells (data are representative of 11 donors). (**E**) Frequency of annexin V^+^ DAPI^+^ tumor cells in 24-hour cocultures with SiRPα monomer–coengineered A97L–T cells at E/T = 1:1, corrected to tumor alone (*n* = 3). (**F**) Schematic of macrophage-mediated tumor cell phagocytosis assay in the presence of SiRPα monomer and cetuximab and/or avelumab. (**G**) Human MDM phagocytosis of A375 tumor cells in the presence of T cell–secreted SiRPα monomer alone versus in combination with cetuximab and/or avelumab (representative results from *n* ≥3 donors). (**H**) NSG murine BMDM phagocytosis of A375 tumor cells in the presence of T cell–secreted SiRPα monomer alone versus in combination with cetuximab and/or avelumab (*n* = 3). Statistical analysis was done by 1-way ANOVA (**E** and **H**) with correction for multiple comparisons by post hoc Tukey’s test (**E** and **H**). *P < 0.05; **P < 0.01; ***P < 0.001; ****P< 0.0001.

We next built retroviral vectors encoding the SiRPα monomers (CV1 and inSiRPα) and GFP (Figure 6A), which achieved high cotransduction (with TCR) of T cells (Figure 6, B and C). We observed CD47 masking on the T cell surface by CV1 (Figure 6D) and noted that constitutive secretion of CV1 did not affect the cytotoxic capacity of the A97L–T cells (Figure 6E). Subsequently, we set up a series of coculture assays comprising macrophages and tumor cells along with CV1 or inSiRPα (collected from culture supernatants of engineered T cells) and the different tumor-specific Abs (Figure 6F). We observed significant increases in A375 phagocytosis by human MDMs in the presence of cetuximab and/or avelumab along with CV1 as compared with CV1 alone (Figure 6G). Anti-MCSP Ab did not synergize with CV1, and trastuzumab did not facilitate ADCP of A375 (Supplemental Figure 7C). The phagocytic response to Abs did not appear to correlate with the expression level of the targeted tumor antigen (Supplemental Figure 6B). The same trends were observed for coculture assays comprising Saos-2 (Supplemental Figure 7D), and isotype controls did not impact phagocytosis (Figure 6G, bottom right). In coculture assays of either A375 (Figure 6H) or Saos-2 (Supplemental Figure 7E) with NSG-BMDMs, CV1 potentiated phagocytosis and synergized with cetuximab and/or avelumab (Figure 6H and Supplemental Figure 7E). Finally, although avelumab is a checkpoint inhibitor, in coculture assays it did not influence target cell killing nor IFN-γ production by the T cells (nor did cetuximab; Supplemental Figure 7, F and G).

### Human T cells coengineered to inducibly secrete CV1 are depleted in NSG mice.

**Figure 7:**
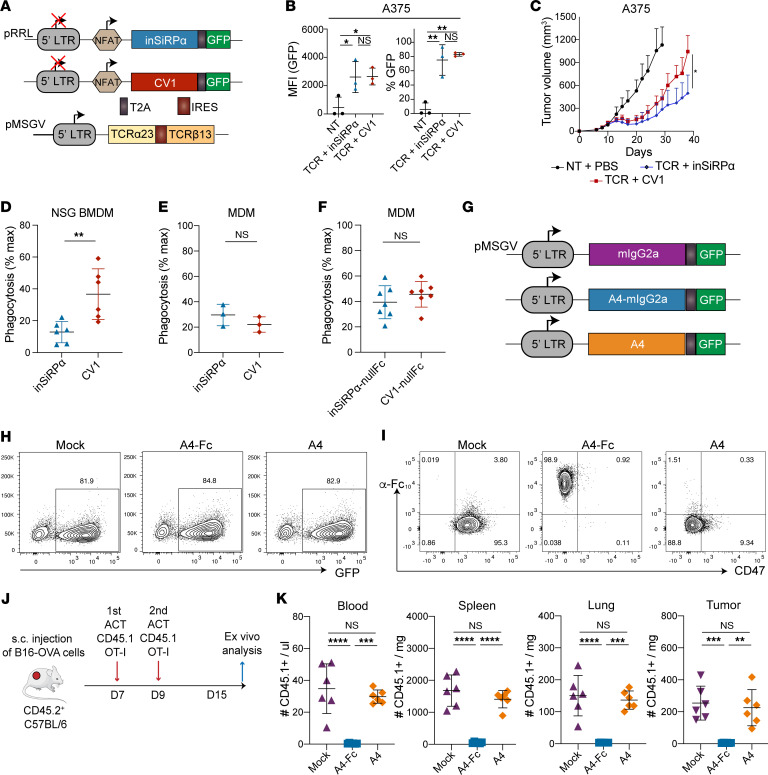
CV1 monomer-coated human T cells are targeted for phagocytosis by NSG but not human macrophages, and engineered murine T cells are only depleted in C57BL/6 mice if the CD47 decoy is fused to an active Fc region. (**A**) Schematic of lentiviral vectors encoding SiRPα monomer under 6xNFAT and of a retroviral vector encoding the A97L-TCR. (**B**) Inducible SiRPα monomer expression by A97L–T cells as detected by EGFP expression upon coculture with target tumor cells (*n* = 3). (**C**) A375 tumor control curves following ACT with A97L–T cells coengineered to express inSiRPα- or CV1 monomers under 6xNFAT (*n* ≥6; data are representative of 2 independent studies). (**D**) Evaluation of phagocytosis of A97L–T cells coengineered to express inSiRPα or CV1 monomers by BMDMs (*n* = 6). (**E**) Evaluation of phagocytosis of A97L–T cells coated with inSiRPα- or CV1 monomers by MDMs (*n* = 3). (**F**) Evaluation of phagocytosis of T cells coated with secreted CV1-nullFc by MDMs in vitro (*n* = 7). (**G**) Schematic of retroviral constructs encoding A4-Fc and A4 decoys. (**H**) Expression of A4-Fc and A4 monomer decoys in transduced mouse OT-I T cells, detected by EGFP (data are representative of 3 or more donors). (**I**) Flow cytometric detection of A4-Fc and A4 monomer binding on OT-I T cells by staining with anti-Fc Ab and anti–mouse CD47 Abs (data are representative of 3 donors). (**J**) Schematic of ACT against subcutaneous B16-OVA tumors and ex vivo analysis. (**K**) Frequency and number of mouse CD45.1^+^ cells in harvested tissues 6 days after ACT (*n* = 6; data are representative of 2 independent studies). Statistical analysis was done by 1-way ANOVA (**B** and **K**), 2-way ANOVA (**C**), or unpaired 2-tailed *t* test (**D**–**F**) with correction for multiple comparisons by post hoc Tukey’s test (**B** and **K**) or post hoc Šidák’s test (**C**). *P < 0.05; **P < 0.01; ***P < 0.001; ****P< 0.0001.

We next built lentiviral vectors encoding CV1 and inSiRPα (monomers) under 6xNFAT (Figure 7A), efficiently cotransduced T cells, and demonstrated decoy expression upon A97L–T cell coculture with target cells (Figure 7B). However, we observed superior A375 tumor control by TCR–T cells expressing inSiRPα (Figure 7C), indicative of T cell depletion upon expression of CV1. Others recently coengineered chimeric antigen receptor (CAR) T cells to secrete a truncated CV1 SiRPα ectodomain (encompassing residues 27–118 versus 27–371) and reported T cell persistence (44), but we observed depletion of T cells coengineered to express this molecule also, albeit at a slower rate (Supplemental Figure 8A).

In subsequent coculture assays, we observed that T cells coengineered to express CV1 (fragment 27–371) were phagocytosed by NSG-BMDMs (Figure 7D) but not human MDMs (Figure 7E). We further demonstrated that human T cells engineered to express CV1 fused to an inactive Fc tail (null) were not phagocytosed by human MDMs (Figure 7F). Thus, our data suggest the potential for the clinical translation of human T cells coengineered to express high-affinity decoys of CD47, provided that they do not comprise an active Fc tail.

We hypothesized that human MDMs do not phagocytose T cells coengineered to express CV1 monomers (or CV1-nullFc) because other “don’t eat me” signals are at play such as CD24 (45), β2m (46), and PD-L1 (47), thus elevating the phagocytic threshold. We further speculated that the persistence of human T cells in NSG mice is critically dependent on human CD47 engagement by NSG-SiRPα on myeloid cells. Indeed, the murine macrophage receptor LILRB1 does not cross-react with human β2m (46), and PD-1 was not present on tumor-infiltrating macrophages nor on neutrophils before ACT (Supplemental Figure 8B). Thus, 2 major “don’t eat me” axes are absent in the xenograft model. However, we did detect Siglec-G (the murine homolog of Siglec-10) on macrophages (Supplemental Figure 8C), which, along with its ligand CD24, constitutes a recently identified “don’t eat me” axis (45). We questioned whether the overexpression of CD24 on human T cells could rescue them from depletion and thus built retroviral vectors encoding murine or human CD24 and the A97L-TCR (Supplemental Figure 8D). We gene-modified human T cells (Supplemental Figure 8E) but found that the overexpression of CD24 did not circumvent CV1-mediated depletion of T cells (Supplemental Figure 8, F and G) and in fact compromised tumor control (Supplemental Figure 8H).

Finally, we sought to test the impact of engineering murine T cells with a monomeric decoy of CD47 versus one fused to an active Fc region, predicting that with other “don’t eat me” axes at play only the latter would lead to murine T cell depletion in C57BL/6 mice. We built retroviral constructs encoding a previously described nanobody A4 targeting CD47, fused or not to mouse IgG2a (mIgG2a) (Figure 7G) (27). We did not use CV1 as it has been reported to have a weaker affinity for murine than for human CD47 (27). Murine OT-I T cells were efficiently transduced with the different constructs (Figure 7H), and expression was detected by flow cytometry (Figure 7I). The engineered T cells were adoptively transferred into C57BL/6 mice bearing subcutaneous B16-OVA tumors (Figure 7J), and while A4-Fc^+^ T cells were depleted in the blood, spleen, lung, and tumor, the A4 monomer^+^ T cells persisted (Figure 7K). These data further support our assertion that there is potential for the clinical translation of tumor-redirected T cells expressing monomeric but not Fc-fused decoys blocking CD47.

### Cetuximab and avelumab cooperate with A97L–T cells to reprogram the TME and improve tumor control.

**Figure 8:**
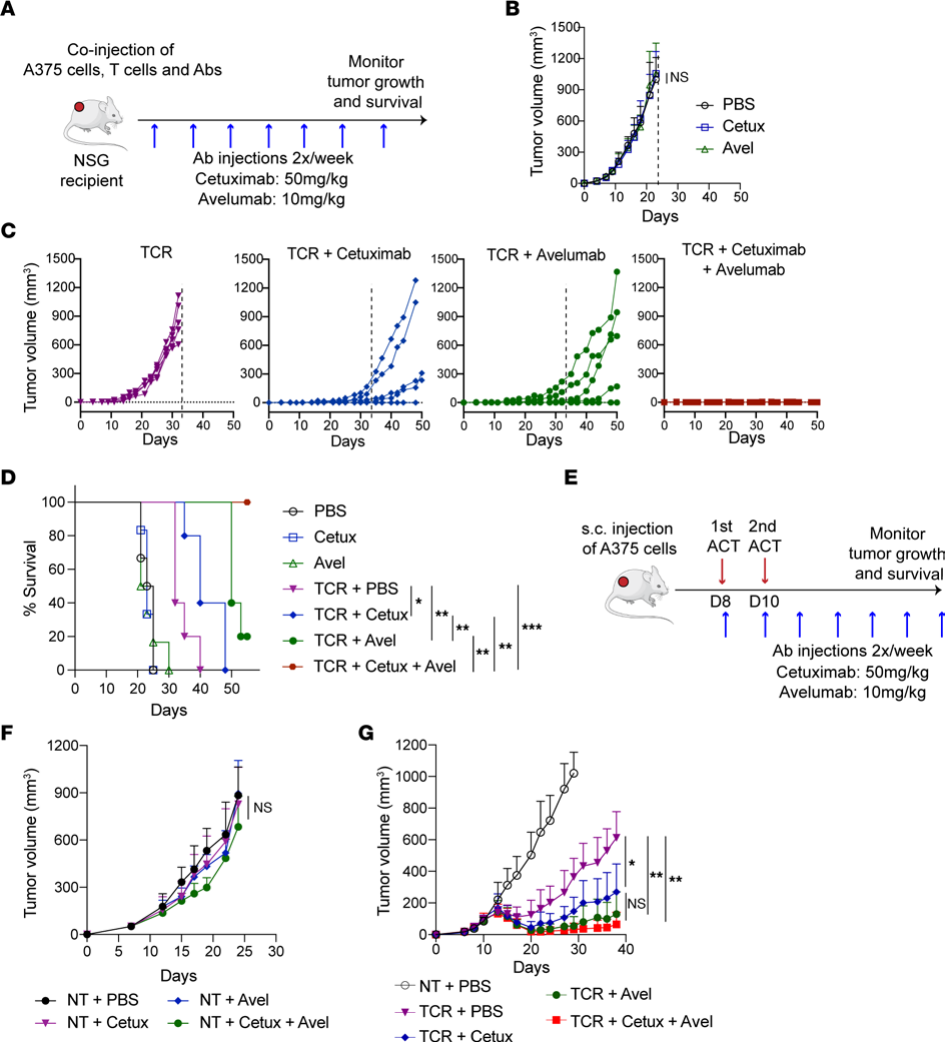
Enhanced tumor control upon coadministration of cetuximab and avelumab with A97L-T cells. (**A**) Schematic of Winn assay. (**B** and **C**) Winn assay with tumor-targeted Abs alone (**B**), with A97L–T cells alone, or for A97L–T cells in combination with cetuximab, and/or avelumab (**C**) (*n* ≥5; data are representative of 2 independent studies). (**D**) Survival curves for the Winn assay. (**E**) Schematic of ACT and Ab coadministration against subcutaneous A375 tumors. (**F**) Treatment of established A375 tumors with Abs alone (*n* = 7; data are representative of 2 independent studies). (**G**) A375 tumor control following ACT with A97L-TCR–T cells alone versus in combination with cetuximab and/or avelumab (*n* ≥5; data are representative of 3 independent studies). Statistical analysis was done by 2-way ANOVA (**B**, **F**, and **G**) or Mantel-Cox (**D**) with correction for multiple comparisons by post hoc Tukey’s test (**B**, **F**, and **G**). *P < 0.05; **P < 0.01; ***P < 0.001; ****P< 0.0001.

While performing the ACT studies described above, we also tested cetuximab and/or avelumab in vivo. We began with Winn assays (Figure 8A) and observed that although Abs alone did nothing (Figure 8B), their coadministration with A97L–T cells further delayed tumor growth and prolonged survival (Figure 8, C and D). Similarly, in a subcutaneous model we observed that the Abs alone had no impact on established tumors but enabled significantly improved control in combination with ACT (Figure 8, E–G). An evaluation of subcutaneous tumor control by A97L–T cells in the presence of full IgG versus F(ab′)_2_ fragments of cetuximab or avelumab revealed a role for the Fc tail in tumor control when targeting EGFR but not PD-L1 (Supplemental Figure 9, A and B).

Ex vivo characterization of tumors (Supplemental Figure 9C) revealed a significant increase in T cell infiltration (Supplemental Figure 9D) upon coadministration of cetuximab and avelumab, as well as a higher CD8/CD4 T cell ratio (Supplemental Figure 9E). Avelumab with or without cetuximab was also associated with higher proliferative capacity of T cells as measured by Ki-67 staining (Supplemental Figure 9, F and G), a higher frequency of central memory T cells (Supplemental Figure 9H), and significantly lower expression of the inhibitory markers PD-L1, TIM-3, and PD-1 on infiltrated T cells (Supplemental Figure 9, I and J). We also observed changes to the endogenous myeloid compartment upon Ab coadministration (Supplemental Figure 10A). For example, we detected higher intratumoral presence of CD11c^+^ DCs, F4-80^+^ macrophages, and F4-80^int^Ly6C^+^ MDMs, but reduced numbers of Ly6G^+^ neutrophils (Supplemental Figure 10, B and C). The macrophages also exhibited a more M1-like phenotype as evaluated by staining for CD38 and Egr2 (Supplemental Figure 10D). Overall, Ab coadministration was associated with changes in the immune microenvironment favorable for supporting tumor control.

Taken together, our data indicate that human T cells gene-modified to secrete high-affinity monomeric decoys of CD47 will not be depleted in treated patients due to other “don’t eat me” signals at play, and that the coadministration of tumor-targeted Abs can favorably reprogram the TME and harness phagocytes against tumor cells for improved clinical outcome.

## Discussion

Although the primary target of immunotherapy is typically adaptive immunity — in particular, cytotoxic T cells — the development of complementary approaches for exploiting innate immunity can help to improve and expand patient responses (9, 12, 48, 49). In addition, strategies for circumventing toxicity, which will likely increase in the context of combination treatments, are critical for advancing efficacious therapies to the clinic (13). Here, with the aim of safely improving the control of solid tumors, we developed a combination ACT strategy to directly increase the activity of human TCR–T cells and harness the phagocytic capacity of endogenous macrophages against tumor cells.

Macrophages play an important role in eliminating tumor cells by phagocytosis, but in most cancers high macrophage infiltration is associated with poor survival. Because macrophages can reversibly alter their endotype in response to environmental cues, such as from parenchymal and immune cells and the extracellular matrix (50), they represent a promising target in cancer immunotherapy. In our study, we focused on blockade of the CD47/SiRPα axis as it has been shown to constitute a powerful innate immune checkpoint in many tumors (51). Our approach may further benefit by combining treatments that modulate the myeloid compartment, such as low-dose irradiation (9) and colony-stimulating factor 1 receptor (CSF1R) inhibitors (52) that can drive tumor infiltration and/or polarize macrophages from an antiinflammatory M2 toward a tumoricidal M1 endotype (53).

Clinical failures due to severe hemolytic reactions caused by hemagglutination after systemic administration of anti-CD47 Ab have been reported (54–56). Although intratumoral Ab delivery may circumvent toxicity (57), this route is unsuitable for metastatic cancers. Hence, at the outset we sought to utilize T cells as a vehicle for delivering decoys that block CD47 directly in tumors. We began by generating T cells gene-modified to express an affinity-optimized A2/NY-TCR (A97L) (30, 31) and observed higher proliferative capacity and presence in A375 melanoma tumors following ACT, as well as significantly improved tumor control and survival compared with WT. We subsequently cotransduced A97L–T cells and demonstrated that secreted CV1-Fc significantly improved macrophage-mediated phagocytosis of tumor cells in vitro as well as control of tumor outgrowth in Winn assays. However, we found that the T cells were themselves coated by CV1-Fc and susceptible to phagocytosis by both human MDMs and mouse BMDMs in vitro, and were depleted upon ACT in NSG mice. Importantly, CV1 monomer– and CV1-nullFc–engineered human T cells were spared from phagocytosis by human MDMs in vitro, presumably because of other “don’t eat me” signals at play.

It has been well established that although CD47 blockade can lower the phagocytic threshold, prophagocytic (“eat me”) signals, such as via Fc/FcR engagement, are needed to drive tumor cell engulfment by macrophages (23, 28). We identified avelumab and cetuximab, both comprising active Fc tails, as capable of binding to human melanoma A375 and cooperating with CV1 (monomer) to augment tumor cell phagocytosis by macrophages. Both Abs also significantly improved subcutaneous A375 tumor control and reprogrammed the TME upon ACT with A97L–T cells. Notably, a growing body of clinical data supports the combinatorial use of cetuximab and avelumab (58). For example, treatment of metastatic colorectal cancer patients with cetuximab drives CD8^+^ T cell infiltration as well as upregulation of PD-L1, the latter of which can be blocked by avelumab (59).

Our data demonstrate a critical role for a cross-species CD47/SiRPα axis in enabling human T cell engraftment in NSG mice because other “don’t eat me” axes either are not present or are not cross-reactive (45–47). Dacek et al., however, reported that they could augment anti-CD20 (rituximab) therapy with CV1-coengineered CD19-CAR-T cells, which persisted in vivo (44). An important difference between our studies is the use of a TCR versus CAR, but others have recently confirmed that CD47 blockade or its downregulation results in human CAR-T cell depletion in vivo (60, 61). A second difference is that Dacek et al. used a smaller region of the CV1-SiRPα ectodomain, which we tested and found led to a slower depletion of T cells in vivo.

In a syngeneic model, coengineering of murine CAR-T cells to secrete SiRPα-Fc was reported by Chen et al. to enhance MC38 tumor control and survival (62). In contrast, we observed rapid depletion of OT-I T cells gene-modified to produce the high-affinity CD47 decoy A4-Fc (27) in B16-OVA–bearing C57BL/6 mice. Importantly, however, we observed that OT-I T cells secreting monomeric A4 persisted in vivo. This demonstrates that with other active “don’t eat me” signals at play, simple blockade of CD47 is insufficient to drive phagocytosis of T cells; a linked “come eat me” signal, such as provided by an active Fc tail, is also needed to break the phagocytic threshold. Notably, in syngeneic models of CD47/SiRPα blockade, either by injected Abs (57) or coengineered T cells, tumor control (e.g., of MC38) can also be supported by “renewable” infiltration of endogenous T cells.

One may speculate that human T cells engineered to secrete decoys of CD47 augment tumor control and persist upon ACT in one in vivo study (or at least a proportion of them do) but not in another study because of the amount of decoy secreted, the affinity of the decoy and Fc region used, the level of CD47 on the T cells and the extent of blockade (57), the proliferative capacity of the T cells, and differences in the phagocytic threshold of the endogenous macrophages. Indeed, we observed a therapeutic window of opportunity for CV1-Fc decoy–coengineered TCR–T cells in our in vivo Winn assays. A shortcoming in our study was our inability to test the combination of CV1-coengineered TCR–T cells targeting A2/NY along with avelumab and/or cetuximab in subcutaneous tumor models because the engineered T cells were depleted in NSG mice, even if the decoy was expressed under an activation-inducible promoter. In vivo testing of this strategy would require transgenic NSG mice (or another immunocompromised strain) in which the endogenous phagocytic cells are responsive to and regulated by other “don’t eat me” signals (45) found on human T cells to elevate the phagocytic threshold.

T cells, along with virtually all other healthy hematopoietic cells, endothelial and epithelial cells, and fibroblasts, express CD47 as a natural mechanism to deter unwanted/unnecessary phagocytosis (15). Here, we have demonstrated that human T cells gene-modified to secrete CV1-Fc but not CV1 are susceptible to phagocytosis by human macrophages. Presumably in clinical studies, systemic monoclonal Ab blockade of CD47, such as with magrolimab, which comprises an active Fc tail, can cause depletion of T cells and other immune cells in treated patients. This may not only increase the risk of infection, but also limit immune-mediated tumor control. Since 2022, multiple phase III clinical trials with magrolimab have been either discontinued or put on hold (ClinicalTrials.gov NCT04313881, NCT04778397, NCT05079230) owing to a lack of survival benefit (compared with standard care for acute myeloid leukemia and myelodysplastic syndromes) or adverse effects. Moreover, solid tumor studies of magrolimab have been placed on partial clinical hold by the Food and Drug Administration. For the phase III ENHANCE-3 study (NCT04313881; announced discontinued in February 2024) an independent data monitoring committee found that magrolimab in combination with azacitidine plus venetoclax demonstrated an increased risk of death (primarily due to infections and respiratory failure) and no benefit.

Taken together, our findings, along with the above-mentioned clinical failures, underscore the challenges in targeting the CD47/SiRPα axis for cancer immunotherapy. We propose a strategy in which decoys of CD47 that are not directly linked to an active “eat me” signal are delivered by gene-modified T cells directly in tumors and appropriate tumor antigen–specific Ab(s) bearing an active Fc tail are coadministered to lower the phagocytic threshold against the tumor cells. In our study we have shown that avelumab (anti–PD-L1) and cetuximab (anti-EGFR) enhance macrophage-mediated phagocytosis of target tumor cells in the presence of CV1 monomer. Moreover, coadministration of these Abs boosts tumor control upon ACT and is associated with favorable changes to the immune microenvironment. It will take extensive preclinical optimization and careful dose escalation studies to safely deliver combinations of ACT with checkpoint blockade of both adaptive and innate immunity as explored in our study, but the strategy holds considerable potential for tackling incurable cancers requiring aggressive treatments.

## Methods

### Sex as a biological variable.

Our study included both male and female mice. Sex-related differences in biological outcome of the therapeutic interventions were not observed. All human T cell donors were anonymous (i.e., ethnicity, age, and sex are unknown).

### Study design.

This study aimed to develop a combination ACT therapy comprising affinity-optimized A2/NY-TCR–T cells coengineered to secrete high-affinity SiRPα decoys to block CD47 in tumors and thereby harness endogenous macrophages and augment tumor control. The decoys, comprising WT, inactive, and high-affinity variants of the SiRPα ectodomain, were first engineered in T cells as Fc fusion proteins, then expressed under 6xNFAT to allow activation-inducible secretion, and finally expressed as monomers combined with tumor-targeting Abs bearing an active Fc tail. Experiments in NSG mice were performed with 5–9 mice per group (as indicated in the figure legends) based on previous experiments showing that this size could guarantee good reproducibility and statistically significant differences. Tumor-bearing mice were randomized into treatment groups before T cell infusion based on the size of the established tumors. Mice were treated by an operator who was blinded to treatment groups. All in vitro experiments were performed with T cells from a minimum of 3 independent healthy donors. The number of repetitions is indicated in the figure legends. All in vitro and in vivo data analysis was based on objectively measurable data. T cell numbers were normalized for all in vitro and in vivo studies based on transduction efficiency with nontransduced T cells added.

### Mice.

NOD.Cg-*Prkdc^scid^* *Il2rg^tm1Wjl^*/SzJ (NSG) mice were obtained from The Jackson Laboratory and subsequently maintained and bred in-house under specific and opportunistic pathogen–free (SOPF) conditions at the Epalinges/UNIL Animal Facility. CD45.1^+^C57BL/6 female mice were also bred in-house, while female CD45.2^+^C57BL/6 mice aged 6–12 weeks were purchased from Harlan (Netherlands).

### Cell lines.

The cell lines A375 (melanoma; HLA-A*0201^+^, NY-ESO-1^+^), NA8 (melanoma; HLA-A*0201^+^, NY-ESO-1^–^), Saos-2 (osteosarcoma; HLA-A*0201^+^, NY-ESO-1^+^), Jurkat (T cell leukemia), HEK293T (human embryonic kidney), and Phoenix-ECO were purchased from ATCC. Me275 (melanoma; HLA-A*0201^+^, NY-ESO-1^+^) was provided by Daniel Speiser (UNIL). CD47-deficient Jurkat-variant JinB8 cells, originally described by Eric Brown, were provided by David Roberts (Center for Cancer Research, National Cancer Institute, Bethesda, Maryland, USA) (63). The B16-OVA melanoma tumor cell line was a gift from Pedro Romero (UNIL). NA8, Me275, A375, and Saos-2 cell lines were engineered with NucLight lentivirus (IncuCyte) to stably express nuclear-restricted mKate2 fluorescent protein in order to track their activity in vitro, according to the manufacturer’s instructions. All human melanoma cell lines were maintained in IMDM (Thermo Fisher Scientific) supplemented with 10% FCS and 1% penicillin/streptomycin. All remaining cell lines were cultured in RPMI plus Glutamax (Thermo Fisher Scientific) supplemented with 10% FCS and 1% penicillin/streptomycin. All cell lines tested negative for mycoplasma contamination.

### Molecular cloning.

The cDNA sequences of TCRs A2/NY-WT, A97L (30, 64), and A2/Melan-A_26–35_ (32) were codon-optimized and synthesized by GeneArt (Thermo Fisher Scientific) in a single cassette separated by IRES or a 2A self-cleaving peptide sequence. To create SiRPα-Fc fusion soluble proteins, the cDNA sequences of the full extracellular domain (aa 27–371) of human WT allele 1 SiRPα (wtSiRPα) cDNA (UniProt: P78324), CV1 (28), and inSiRPα (34) were codon-optimized and synthesized by GeneArt (Thermo Fisher Scientific), fused with the cDNA sequence of the human hinge and IgG1 heavy chain Fc region (pFUSE-hIgG1-Fc1, InvivoGen), downstream of a mouse Ig κ chain secretion signal peptide. Proteins were produced in CHO cells and purified by affinity chromatography. For the A4-Fc fusion proteins, the cDNA of the nanobody (27) was codon-optimized and synthesized by GeneArt (Thermo Fisher Scientific), fused with the cDNA sequence of the mouse hinge and IgG2a heavy chain Fc region (pFUSE-mIgG2a-Fc1, InvivoGen), downstream of a mouse Ig κ chain secretion signal peptide. Subsequently, the cassettes were cloned into pRRL lentiviral (under the control of a human PGK promoter), pMSGV retroviral, or pXLG expression (under the control of a CMV promoter) vectors, followed by the enhanced GFP (EGFP) reporter gene where noted, using standard molecular cloning techniques. For inducible expression in T cells, the sequences of all monomer and Fc-fused SiRPα molecules were subcloned into a pRRL lentiviral vector under the control of 6xNFAT as previously described (65). To generate an anti–human MCSP/CSPG4 hIgG1-Fc fusion Ab-like protein, the sequence of the light chain of the anti–human MCSP/CSPG4 scFv (clone 9.2.27) was subcloned into the pXLG expression vector followed by a GS linker and the sequence of the heavy chain. The cassette was placed upstream of the cDNA sequence of the human hinge and IgG1 heavy chain Fc region to generate an IgG1-Fc fusion protein.

### Flow cytometry.

Single-cell suspensions were stained with Abs and matched isotypes (Supplemental Table 1). SiRPα-Fc binding on tumor cells was detected by incubation of the cells with either soluble or T cell–secreted SiRPα-Fc at 4°C for 1 hour, followed by staining with labeled goat anti-human IgG-Fc at 4°C for 30 minutes. To determine binding avidity, soluble SiRPα-Fc immobilized on 0.4–0.6 μm Protein G Yellow Fluorescent Particles (PGFP-0552-5, Spherotech) was incubated with tumor cells at 4°C for 1 hour. Cell binding was assessed by detection of the fluorescent beads. Binding of T cell–secreted A4-Fc on OT-I T cells was assessed by staining with labeled goat anti–mouse IgG-Fc (polyclonal, Antibodies-online) at 4°C for 30 minutes. GFP expression was used as a surrogate marker for transduction efficiency. DAPI staining was used to exclude dead cells, and apoptotic cells were excluded by staining with annexin V at 4°C in the dark for 15 minutes. Cell acquisition was performed on an LSRII flow cytometer (BD Biosciences), and data were analyzed using FlowJo (Tree Star). For detection of intranuclear Ki-67, cells were fixed and permeabilized with the FoxP3 transcription factor staining buffer set (Thermo Fisher Scientific) at 4°C for 1 hour and subsequently stained with an anti–human Ki-67 (Ki-67, BioLegend) Ab at room temperature in the dark for 45 minutes.

### Production of retrovirus and lentivirus.

To produce lentiviral particles, HEK293T cells were cotransfected with 15 μg pRRL transfer plasmid and 7 μg pVSV-G and 18 μg R874 (containing Rev and Gag/Pol) lentiviral packaging plasmids. For the production of retroviral particles, HEK293T cells were cotransfected with 21 μg pMSGV transfer plasmid and 18 μg pMD22-Gag/Pol and 7 μg pMD RD114 (feline endogenous virus envelope glycoprotein) retroviral packaging, using a mix of Opti-MEM medium (Thermo Fisher Scientific) and Turbofect (Thermo Fisher Scientific). To produce mouse ecotropic retroviral particles, Phoenix-ECO cells were cotransfected with 21 μg pMSGV transfer plasmid and 14 μg pCL-ECO retroviral packaging plasmid. Culture supernatants were collected 48 hours and/or 72 hours after transfection and concentrated by ultracentrifugation at 24,000*g* for 2 hours. The concentrated virus was stored at –80°C until use. Viral titers and MOIs were determined by EGFP reporter gene expression in HEK293T cells.

### Human and mouse T cell isolation, stimulation, viral transduction, and expansion.

Buffy coats were purchased from Transfusion Interrégionale CRS SA (Epalinges, Switzerland), PBMCs prepared using Lymphoprep (Axis-Shield), and T cells isolated by magnetic microbeads (Miltenyi Biotec or StemCell Technologies). Both human and splenic CD45.1^+^ OT-I T cells were isolated using kits (StemCell Technologies) and stimulated with anti-CD3/CD28 beads (Thermo Fisher Scientific) at a 2:1 bead/T cell ratio in the presence of 50 IU/mL human IL-2 (GlaxoSmithKline). Lentiviral transduction of human T cells was performed 24 hours after activation by addition of viral particles in the culture medium (MOI 20) in the presence of Lentiboost (Sirion Biotech). Retroviral transduction of T cells was performed 48 hours after activation. Briefly, T cells were transferred to retronectin-coated (Takara) plates previously spinoculated with retroviral particles at 2,000*g* for 1.5 hours. T cells were removed from retronectin-coated plates the next day, CD3/CD28 beads removed as indicated (3–5 days after activation), and the T cells maintained in RPMI 1640–Glutamax (Thermo Fisher Scientific) supplemented with 10% heat-inactivated FCS, 1% penicillin/streptomycin, 10 ng/mL human IL-7 and IL-15 (Miltenyi Biotec) at 0.5 × 10^6^ to 1 × 10^6^ T cells/mL. Mouse T cell medium was further supplemented with 1 mM sodium pyruvate, 50 μM β-mercaptoethanol, and 10 mM nonessential amino acids. T cells were typically counted manually every 2–3 days using 0.1% trypan blue staining (Thermo Fisher Scientific). Transduction efficiency was determined on day 7. For NFAT reactivation, T cells were stimulated for 48 hours with PMA/ionomycin (Cell Stimulation Cocktail, Thermo Fisher Scientific) or PHA (MilliporeSigma).

### IFN-γ production and T cell cytotoxicity assays by flow cytometry.

Briefly, 10^5^ rested NY-ESO-1 TCR^+^ T cells (4:1 CD8^+^/CD4^+^) were cocultured with 10^5^ NucLight red^+^ tumor cells in complete medium for 24–48 hours. IFN-γ levels in collected cell-free culture supernatants were determined by ELISA (Thermo Fisher Scientific). T cell cytotoxicity, determined by flow cytometry analysis of the cells, was defined as the percentage of annexin V^+^/DAPI^+^ tumor cells. Results were normalized to the percentage of annexin V^+^/DAPI^+^ in cultures of tumor cells alone.

### T cell cytotoxicity assay by IncuCyte live-cell imaging.

For the assay, 1.5 × 10^4^ rested NY-ESO-1 TCR^+^ T cells (4:1 CD8^+^/CD4^+^) were cocultured with 1.5 × 10^4^ NucLight red^+^ tumor cells in complete medium for up to 96 hours. Phase and red fluorescence images were acquired every 2 hours using IncuCyte ZOOM (Essen Biosciences). Tumor cell growth was determined by following Total Red Object Area/mm^2^ over time.

### T cell proliferation assay.

For the assay, 10^5^ rested A2/NY-TCR^+^ T cells (4:1 CD8^+^/CD4^+^) were labeled with 0.5 μM CFSE (Thermo Fisher Scientific) and cocultured with 10^5^ NucLight red^+^ tumor cells in complete medium. T cell proliferation was assessed 5 days later by flow cytometry and was defined as the percentage of live CFSE^–^ T cells in the culture. T cells activated with PMA/ionomycin (Thermo Fisher Scientific) were used as a positive control.

### Production of human SiRPα-Fc molecules by human T cells.

CD8^+^ and CD4^+^ T cells were cultured separately in serum-free medium supplemented with 10 ng/mL IL-7/IL-15 at a concentration of 10^6^ SiRPα-engineered T cells/mL for 24, 48, 72, and 96 hours. Human SiRPα-Fc levels in supernatants were determined by an in-house ELISA. Briefly, collected supernatants were incubated with immobilized human CD47 (ACROBiosystems). Bound SiRPα-Fc was detected with a secondary biotinylated rabbit anti–IgG-Fc Ab (Thermo Fisher Scientific) followed by HRP-conjugated streptavidin (Thermo Fisher Scientific). Soluble CV1-SiRPα-Fc of known concentration was used as a standard.

### Generation of human CD14^+^ MDMs.

CD14^+^ monocytes were positively isolated from PBMCs using CD14 magnetic microbeads (Miltenyi Biotec). Macrophages were generated by culturing of isolated CD14^+^ monocytes in RPMI supplemented with 10% FCS, 1% penicillin/streptomycin, and 50 ng/mL human M-CSF (ImmunoTools) for 7 days and harvesting of the adherent fraction. The medium was refreshed on days 3 and 6.

### Generation of mouse BMDMs.

Whole bone marrow cells were isolated by flushing of the femora and tibiae of NSG mice. Macrophages were generated by incubation of whole bone marrow cells in DMEM plus Glutamax (Thermo Fisher Scientific) supplemented with 10% FCS, 1% penicillin/streptomycin, 50 μM β-mercaptoethanol, and 50 ng/mL mouse M-CSF (ImmunoTools) for 7 days and harvesting of the adherent fraction. The culture medium was refreshed on days 3 and 6.

### In vitro Ab-dependent cellular phagocytosis assays.

Briefly, 5 × 10^4^ human MDMs or murine (NSG) BMDMs were cocultured with 5 × 10^4^ PKH26-labeled (MilliporeSigma) tumor cells in serum-free medium in 96-well ultra-low adherent plates (Corning) and incubated for 4–6 hours at 37°C. To test human T cell–secreted SiRPα-Fc and SiRPα monomer molecules, either 5 × 10^4^ transduced T cells were added to the macrophage/tumor coculture or the macrophage/tumor culture was performed in supernatants harvested from 24-hour decoy-engineered T cell cultures. As indicated, recombinant human SiRPα-IgG1-Fc fusion proteins, anti-CSPG4 hIgG1-Fc, cetuximab (Erbitux, Merck), trastuzumab (Herceptin, Roche), avelumab (Bavencio, Merck), or a matched isotype were added at 10 μg/mL. At the end of coculture, the cells were washed twice and incubated with human or mouse Fc receptor Abs (BD Biosciences). Human macrophages were stained with anti-CD11b and anti-CD64 Abs. Murine macrophages were stained with anti-CD11b and anti-F4/80 Abs. Phagocytosis was determined as the percentage of PKH26^+^ cells within human CD11b^+^CD64^+^ and mouse CD11b^+^ F4/80^+^ macrophages, respectively.

### Amnis ImageStream imaging of tumor cell phagocytosis.

5 × 10^4^ human MDMs or murine (NSG) BMDMs were cocultured with 5 × 10^4^ PKH26-labeled (MilliporeSigma) tumor cells in the presence of the different recombinant SiRPα decoys at 10 μg/mL in serum-free medium in 96-well ultra-low adherent plates (Corning) and incubated for 4–6 hours at 37°C. Samples were run in the Amnis ImageStreamX multispectral imaging flow cytometer (Cytek), and images of 15,000 events were acquired per sample. Cells were analyzed using a 405 nm laser (15 mW) for DAPI excitation, a 488 nm laser (100 mW) for PKH26 dye excitation, and a 642 nm laser (130 mW) for APC excitation. Bright-field, side scatter, and fluorescent cell images were acquired at ×40 magnification. Each experimental file contained imagery for 10,000 cells, with each cell analyzed for bright field, side scatter (SSC; Ch06), and 3 fluorescence channels (DAPI nuclear stain, PKH26 dye, and anti–human CD11b–APC). Single-color controls were acquired to generate a compensation matrix that was applied to all the experimental files before analysis using IDEAS 6.2 software. Only events with APC areas greater than 60 μm^2^ to exclude cell debris and non-saturating pixels were collected as previously described (66). Images of DAPI^–^APC^+^PKH26^+^ double-positive events were analyzed downstream with IDEAS 6.2 software. To determine phagocytosis of tumor cells by macrophages, we measured the distance between the centers of the PKH26 and APC images of DAPI^–^APC^+^PKH26^+^ double-positive cells using the Delta Centroid XY (DC) feature. Events in which macrophages have phagocytosed target/tumor cells have lower DC values than aggregate events.

### Winn assay.

NSG mice aged 6–12 weeks were subcutaneously inoculated on the flank with 3 × 10^6^ A375 or Me275 tumor cells previously mixed with 6 × 10^6^ (or as otherwise indicated) A2/NY-TCR–T cells coengineered or not to secrete different SiRPα decoys as indicated (4:1 CD8^+^/CD4^+^). Where indicated, 1 mg cetuximab and/or 0.2 mg avelumab were administered intraperitoneally twice per week. Start of treatment coincided with T cell injection, and treatment continued throughout the experiment unless otherwise indicated. Tumor growth was monitored by caliper measurements 2–3 times per week, and tumor volumes were calculated using the formula *V* = ½(*L* × *W*^2^), where *L* is the greatest longitudinal diameter and *W* is the greatest transverse diameter. Mice were sacrificed when tumors reached 1,000 mm^3^ or when the mice lost more than 20% of their original weight or became weak and moribund. Each group consisted of at least 5 mice.

### ACT in xenograft and syngeneic tumor models.

Female NSG mice aged 6–12 weeks were subcutaneously inoculated on the flank with 10^6^ A375 melanoma cells. Male NSG mice aged 6–12 weeks were subcutaneously inoculated on the flank with 5 × 10^6^ Me275 cells. T cells were adoptively transferred when tumors reached 50–100 mm^3^. Mice were treated twice with 10^7^ TCR-expressing SiRPα-Fc– or SiRPα monomer–secreting T cells (4:1 CD8^+^/CD4^+^) or an equivalent number of nontransduced (NT) or mock-transduced T cells, with the second ACT performed 2–3 days after the first one. Where indicated, 1 mg cetuximab and/or 0.2 mg avelumab were administered intraperitoneally twice per week. Cetuximab and avelumab F(ab′)_2_ fragments were generated using the Pierce F(ab′)_2_ preparation kit (Thermo Fisher Scientific) and administered intraperitoneally twice per week at equimolar amounts to intact Ab counterparts, starting at the first T cell transfer. Female C57BL/6 mice aged 6–12 weeks were subcutaneously inoculated on the flank with 10^5^ B16-OVA melanoma cells. T cells were intravenously transferred to mice when tumors reached 50–100 mm^3^. Mice were treated twice with 5 × 10^6^ OT-I A4-Fc– or A4 monomer–secreting T cells, or an equivalent number of NT or mock-transduced T cells, with the second ACT performed 2–3 days after the first one. Tumor growth was monitored, and mice were sacrificed as described above for Winn assays.

### Ex vivo studies.

Mouse tissues were collected at the endpoint as indicated, and organs were weighed. Tumors and lungs were excised, minced, and dissociated in Liberase (Roche) at 37°C for 1 hour. Single-cell suspensions were prepared by mechanical dissociation over a 70 μm strainer (Greiner). Harvested spleens were mechanically dissociated over a 40 μm strainer (Greiner). Blood samples were collected via cardiac puncture at the time of sacrifice. RBCs were depleted (RBC lysis solution, QIAGEN) from single-cell suspensions.

### Statistics.

All statistical analyses were performed on GraphPad Prism 6 software, as indicated in the figure legends. *P* less than 0.05 was considered statistically significant. Mean ± SD was used to summarize the data unless otherwise noted. Statistical differences in means of 2 groups were calculated by 2-tailed parametric Student’s *t* tests for unpaired data. Statistical comparisons in means of 3 groups or more were performed by 1-way ANOVA or 2-way ANOVA with correction for multiple comparisons using Tukey’s test (all groups compared) or Šidák’s test (2 select groups compared). The Kaplan-Meier method was used to generate median survival, statistically analyzed by log-rank test. 

### Study approval.

All in vivo experiments were approved by the Service of Consumer and Veterinary Affairs of the Canton of Vaud and Swiss federal law.

### Data availability.

All raw data are available in the Supporting Data Values file. Data analyses and sequences of constructs presented in the article are available upon request.

## Author contributions

MI directed the study. GC and RS provided advice. ES and MI conceived and planned the experiments. ES, AS, JP, and BS performed the experiments. KS provided early technical advice. OM and VZ developed the A2/NY-TCR with MI. ES, MI, and AS analyzed data. ES, MI, AS, and JP contributed to the interpretation of the results. ES prepared the figures and the first draft of the manuscript. MI revised and, together with ES, rewrote the manuscript and finalized it.

## Supplementary Material

Supplemental data

Supporting data values

## Version 1 06/03/2024

Electronic publication
