# Supplementary material for: Combining SiRPα decoy–coengineered T cells and antibodies augments macrophage-mediated phagocytosis of tumor cells
Source: J Clin Invest. 2024 Apr 23;134(11):e161660. doi: 10.1172/JCI161660 (PMC11142748; doi:10.1172/JCI161660)
Supplement: Supplemental data [file jci-134-161660-s062.pdf]

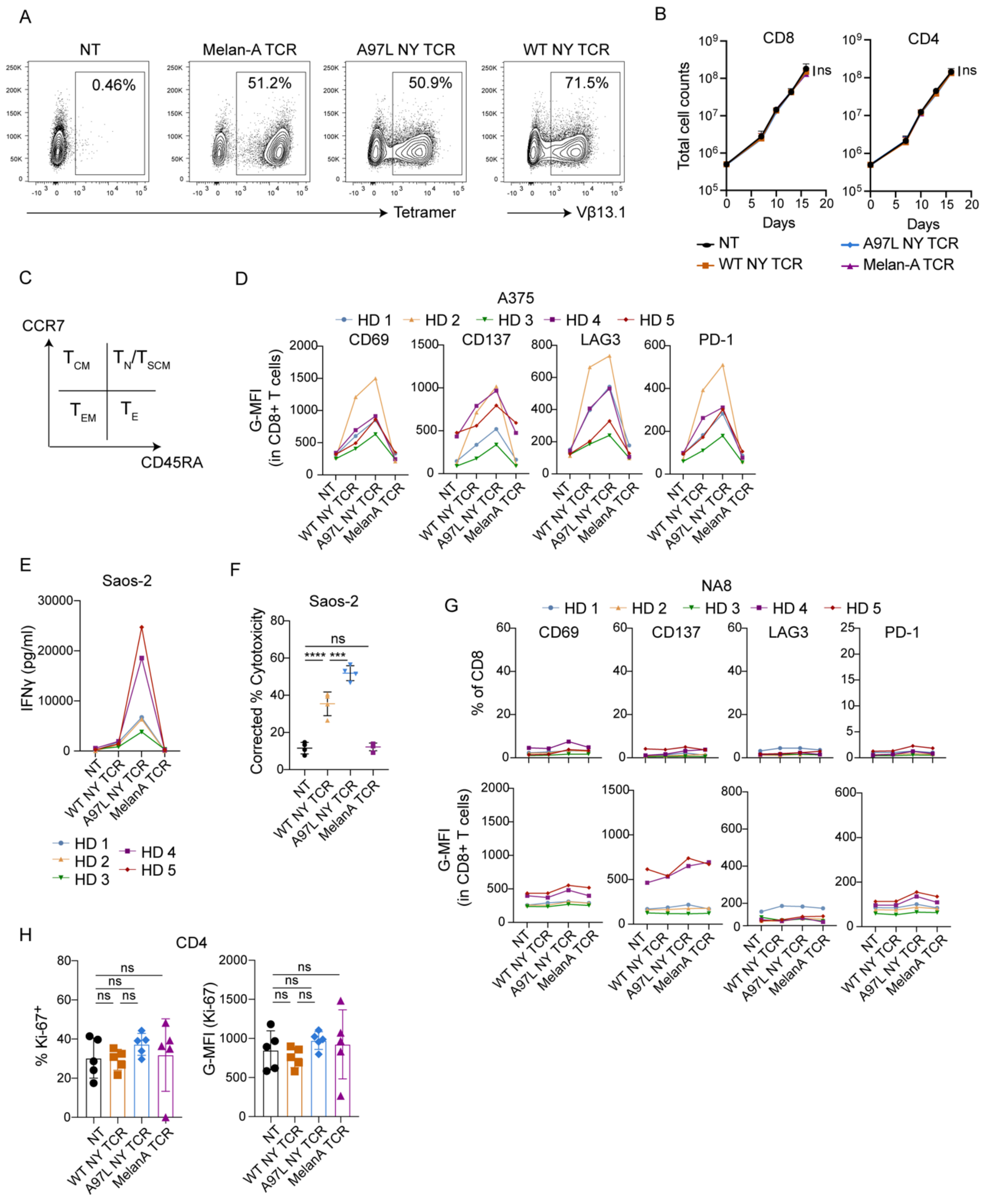

Supplemental Figure 1. Phenotype and function of TCR-engineered T-cells. (A) Transduction efficiency of $CD4^+$ T-cells with TCRs evaluated by tetramer or anti-Vβ13.1 Ab staining (data representative of n=5 donors). (**B)** Total counts of $CD8^+$ and $CD4^+$ T-cells obtained in vitro over 16 days of culture (n=4). (**C)** Staining and gating strategy to evaluate effector and memory phenotypes within cultures of rested T-cells, transduced or not to express a TCR (Effector, T_E; Effector Memory,

T_{EM}; Central Memory, T_{CM}; Naïve/Stem-cell-like Memory, $T_{N/SCM}$). **(D)** Expression (G-MFI) of activation markers and checkpoint receptors on $CD8^+$ T-cells 24h post-stimulation with $A2^+/NY^+$ A375 tumor cells (n=5), HD: Healthy Donor. **(E)** IFNγ secretion levels by TCR-modified T-cells 24h post-stimulation with Saos-2 tumor cells at E:T = 1:1 (n=5), HD: Healthy Donor. **(F)** Frequency of $AnnexinV^+DAPI^+$ cells, corrected to tumor alone, in 24h co-cultures of Saos-2 tumor cells with TCR-T-cells at E:T=1:1 (n=4). **(G)** Expression (frequency and G-MFI) of activation markers and checkpoint receptors on $CD8^+$T-cells 24h post-stimulation with $A2^+/NY^-$ NA8 tumor cells (n=5), HD: Healthy Donor. **(H)** Frequency (left) and G-MFI (right) of Ki-67 expression within intratumoral human $CD4^+$T-cells 7 days post-ACT (n=5, data representative of 2 independent experiments). Statistical analysis by two-way analysis of variance (ANOVA) (B) or one-way ANOVA (F, H) with correction for multiple comparisons by post hoc Tukey's test (B, F and H). ****P< 0.0001; ***P < 0.001; **P < 0.01; *P < 0.05.

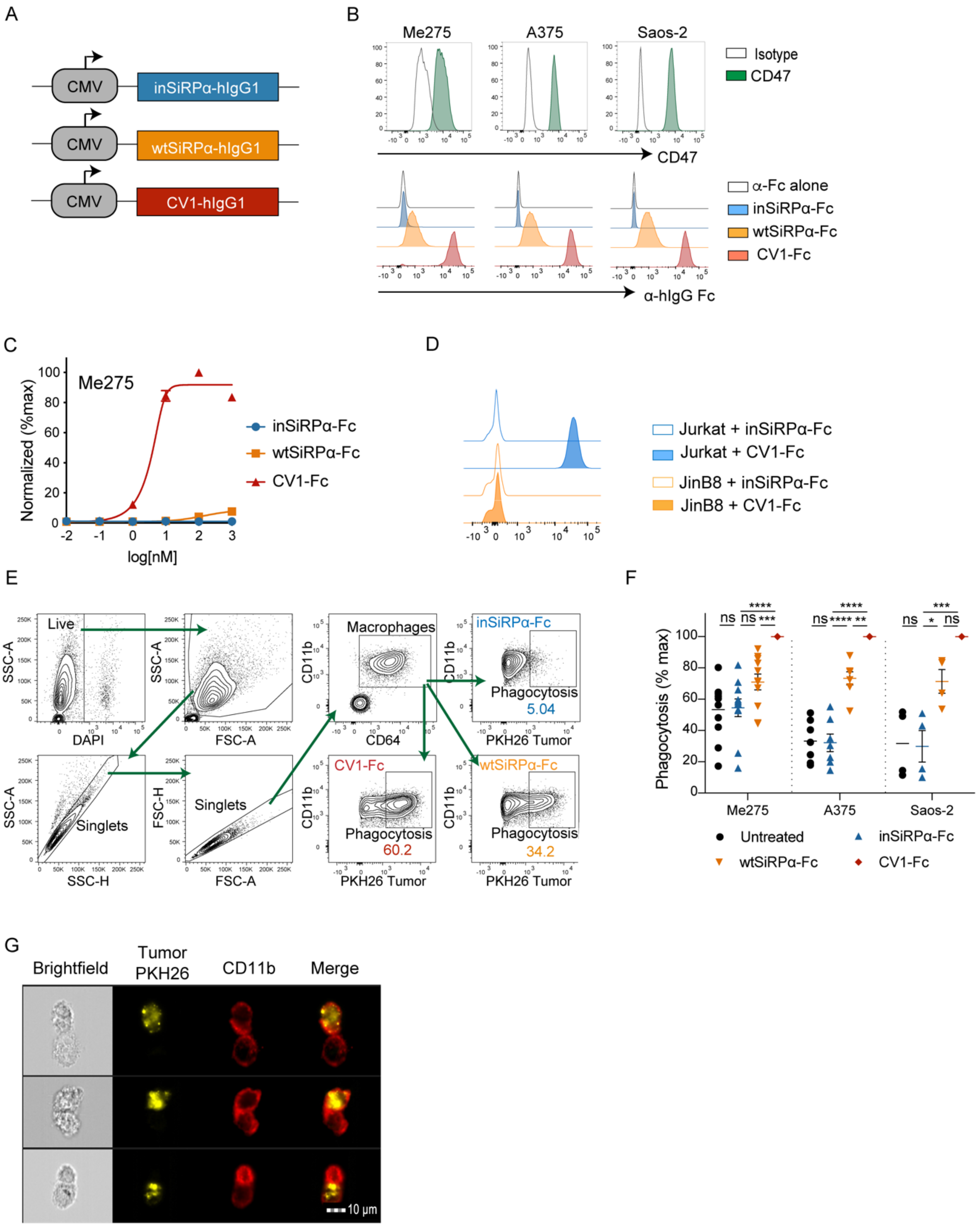

Supplemental Figure 2. Soluble SiRPα-Fc binds CD47 on tumor cells, enabling enhanced phagocytosis by human MDMs. (A) Schematic of expression vectors encoding human inSiRPα-Fc, wtSiRPα-Fc, and CV1-Fc proteins. (**B)** Panel of HLA-A2^{+} NY-ESO-1^{+} tumor cell lines expressing CD47 on their cell surface (top) and detection of wtSiRPα-Fc and CV1-Fc soluble protein binding on tumor CD47 by anti-human IgG Fc Ab staining (bottom). (**C)** Binding of increasing concentrations of SiRPα-Fc to CD47 on Me275 tumor cells (data representative of n=2 independent studies). (**D)** SiRPα-

Fc binding profile on CD47$^+$ Jurkat and CD47-deficient JinB8 cells (data representative of n=3 independent studies). **(E)** Gating strategy for flow cytometric detection of PKH26-labeled tumor cell phagocytosis by human myeloid-derived macrophages (MDMs) in vitro. **(F)** Phagocytosis of PKH26-labeled tumor cells by human MDMs in the presence of 10ug/ml soluble SiRPα-Fc proteins. Phagocytosis is normalized to the maximal response of each individual donor ($n \geq 4$). Data represent mean ± SEM. **(G)** Representative Amnis ImageStream images of human MDMs engulfing PKH26-labeled Saos-2 tumor cells after treatment with 10ug/ml CV1-Fc. Statistical analysis by one-way analysis of variance (ANOVA) (F) with correction for multiple comparisons by post hoc Tukey's test on pooled donors (F). ****$P< 0.0001$;***$P < 0.001$; **$P < 0.01$; *$P < 0.05$.

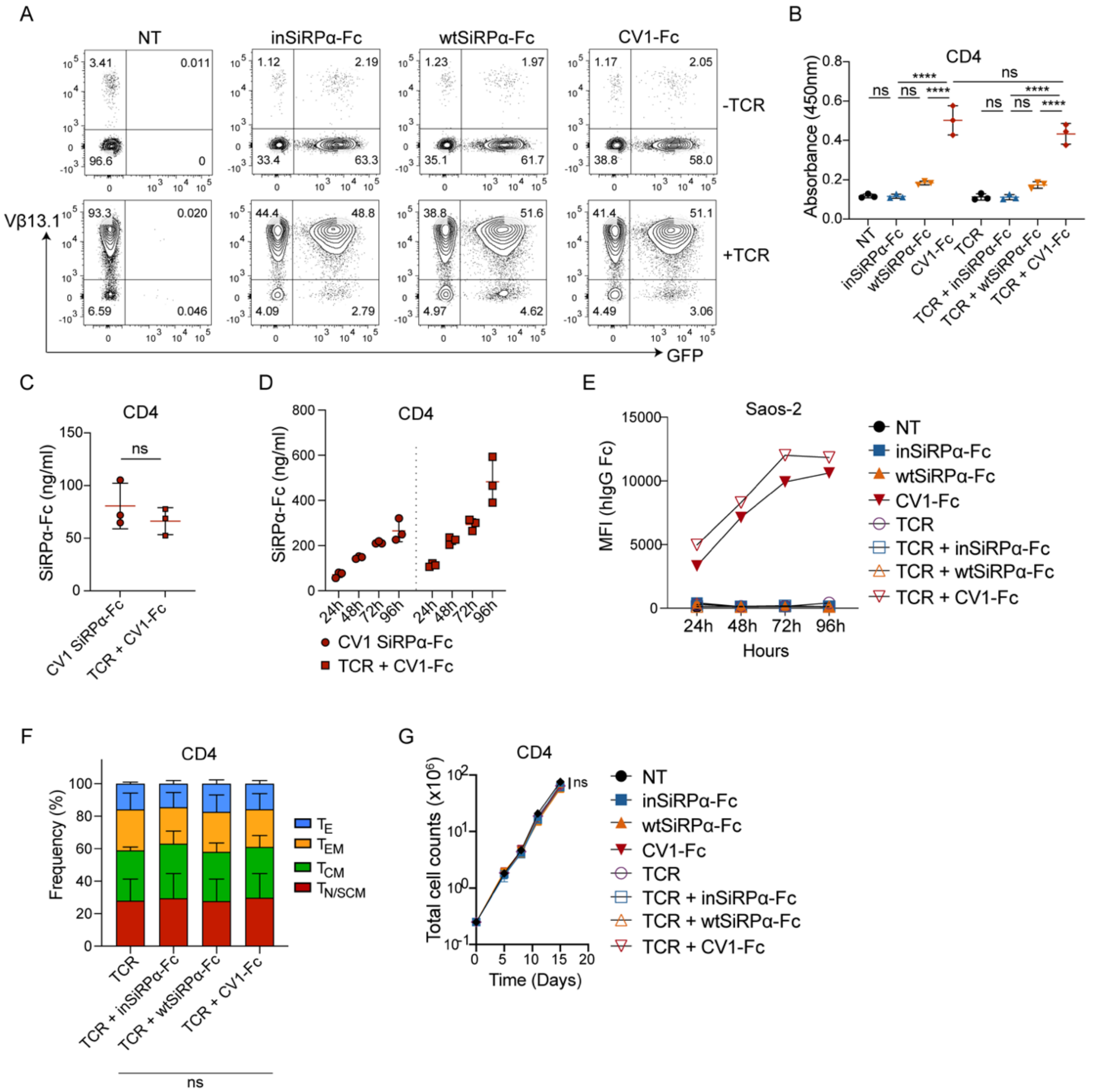

Supplemental Figure 3. SiRPα-Fc binds to CD47 on tumor cells and increases phagocytosis by human macrophages. A) Expression of SiRPα-Fc and A97L TCR in transduced CD4$^+$ T-cells, detected by eGFP and anti-Vβ13.1 Ab staining, respectively (data representative of n=12 donors). **B)** CD47-based ELISA detection of SiRPα-Fc secreted by engineered CD4$^+$T-cells (n=3). **C)** Quantification of CD4$^+$T-cell-secreted CV1-Fc by CD47-based ELISA (n=3). **D)** Quantification of CV1-Fc accumulated in culture supernatants of engineered CD4$^+$ T-cells over time. **E)** Binding of CD8$^+$ T-cell-secreted CV1-Fc on Saos-2 using culture supernatants at 24 to 96 hours (data representative of n=2 independent studies). **F)** Frequency of effector and memory phenotypes of transduced and rested CD4$^+$T-cells (n=3) (Effector, T_E; Effector Memory, T_{EM}; Central Memory, T_{CM}; Naïve/Stem-cell like Memory, $T_{N/SCM}$). **G)** Expansion of engineered CD4$^+$T-cells (n=3). Statistical analysis by one-way analysis of variance (ANOVA) (B and F), unpaired two-tailed t-test (C), or two-way ANOVA (G) with correction for multiple comparisons by post hoc Tukey's test on pooled donors (B and F-G). ****P< 0.0001;***P < 0.001; **P < 0.01; *P < 0.05.

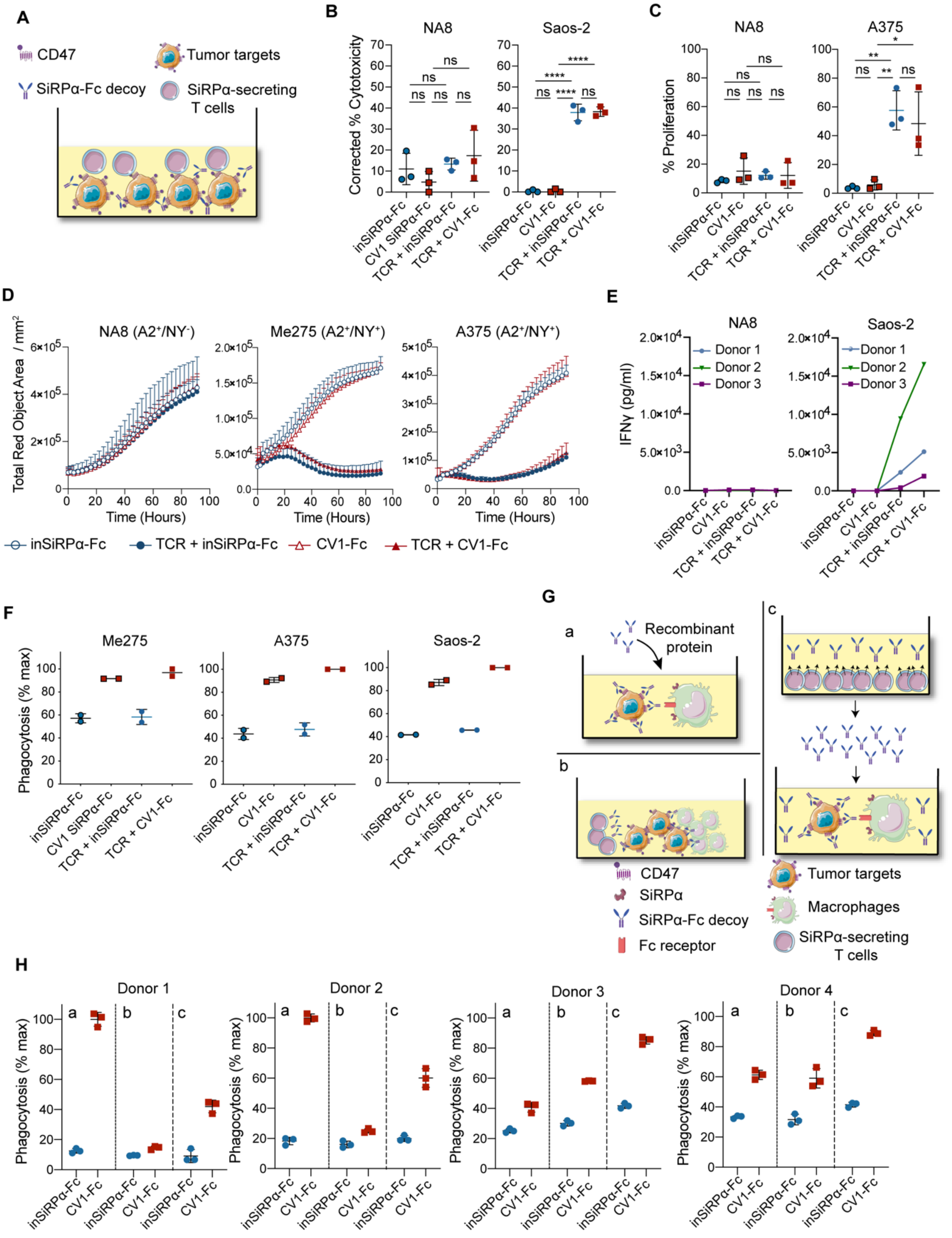

Supplemental Figure 4. Evaluation of engineered T-cell function as well as macrophage-mediated tumor-cell phagocytosis in the presence of CV1-Fc decoys in different co-culture conditions. (A) Schematic of tumor cell and engineered T-cell co-culture to evaluate the impact of decoys on effector

function. **(B)** Frequency of Annexin V$^+$ DAPI$^+$ cells, corrected to tumor alone, 24h post-stimulation with NA8 or Saos-2 tumor cells (n=3). **(C)** Frequency of proliferating engineered CD8$^+$T-cells following stimulation with NA8 or A375 tumor cells (n=3). (**D**) Fluorescent tracking of mKate2$^+$ tumor cells in co-culture with SiRPα-decoy coengineered A97L-T-cells by live-cell Incucyte imaging (data representative of n=3 donors). (**E**) IFNγ production by engineered T-cells 24h post-stimulation with NA8 and Saos-2 tumor cells (n=3 donors). (**F**) Tumor-cell phagocytosis by MDMs in the presence of T-cell-secreted CV1-Fc decoy molecules from single- or TCR dual-transduced T-cell cultures (n=2). **(G)** Schematic of different co-culture conditions to evaluate tumor-cell phagocytosis by macrophages in the presence of CV1-Fc decoy (a = recombinant decoy protein, b = decoy secretion by T-cells in a triple co-culture, c = decoy supernatant). **(H)** Side-by-side comparison of tumor-cell phagocytosis under conditions shown in Fig. S3G for MDMs (n=4). Statistical analysis by one-way analysis of variance (ANOVA) (B-C) with correction for multiple comparisons by post hoc Tukey's test (B-C). ****P< 0.0001; ***P < 0.001; **P < 0.01; *P < 0.05.

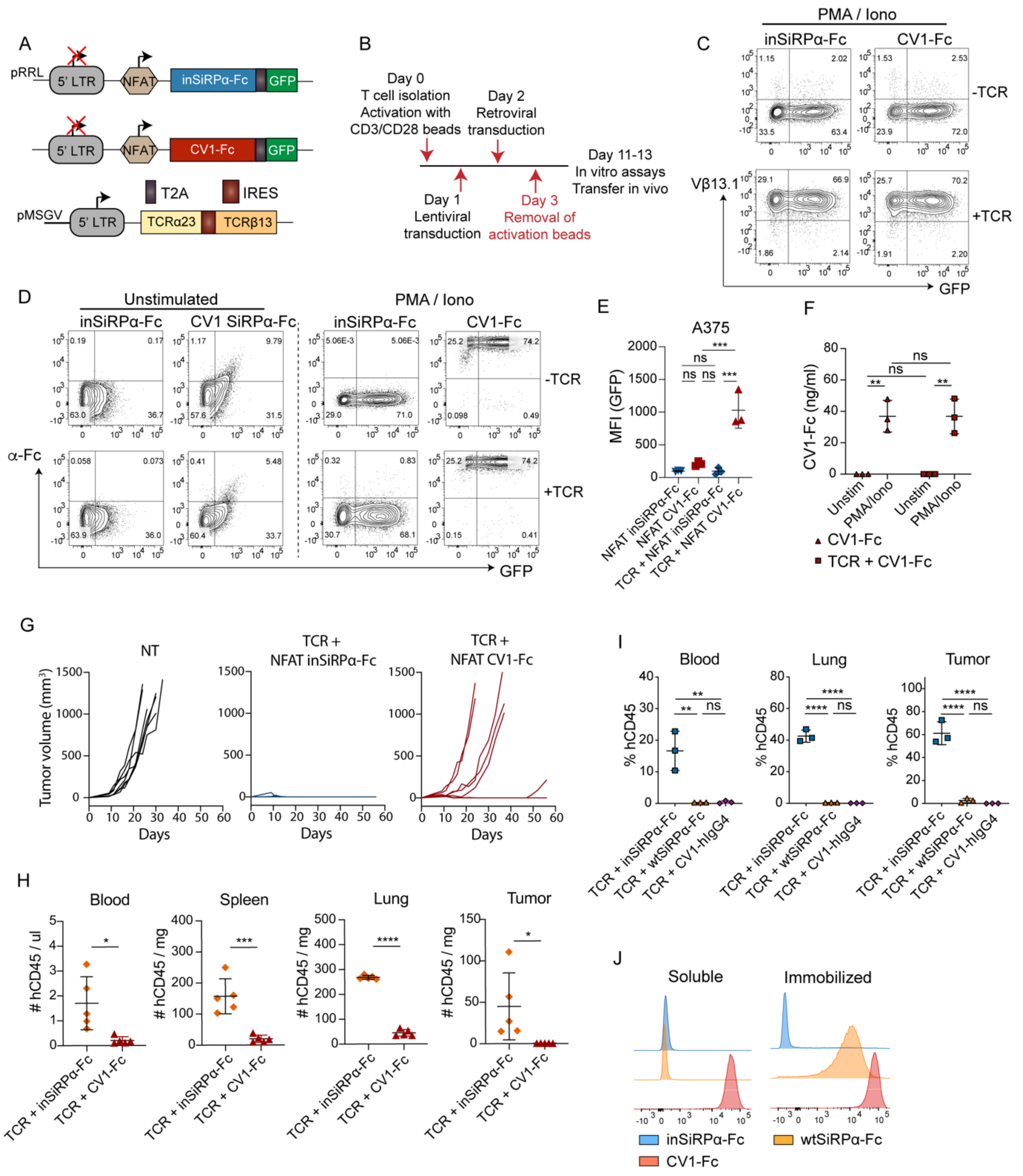

Supplemental Figure 5. SiRPα-decoys expressed under the 6xNFAT promoter are efficiently secreted upon T-cell activation. (A) Schematic of lentiviral constructs encoding SiRPα-Fc decoys under 6xNFAT and of a retroviral construct encoding the A97L-TCR. **(B)** Strategy for optimized T-cell activation, transduction, and expansion to minimize decoy secretion pre-ACT. **(C)** Detection of SiRPα-Fc and A97L-TCR by eGFP and anti-Vb13 Ab staining, respectively, in rested engineered T-cells re-activated with PMA-Ionomycin for 48h (data representative of n=3 donors). **(D)** Secreted CV1-Fc detected on the surface of PMA-Ionomycin activated T-cells versus resting T-cells (data representative

of n=3 donors). Gates have been placed based on non-transduced T-cells. **(E)** Flow cytometric evaluation of CV1-Fc expression upon coculture of engineered A97L-T-cells with target tumor cells (n=3). **(F)** CD47-based ELISA evaluation of CV1-Fc secretion by engineered T-cells upon PMA-Ionomycin activation (n=3). **(G)** Tumor control curves upon ACT with A97L-T-cells expressing SiRPα-decoys under 6xNFAT (n=7). **(H)** Ex vivo evaluation of T-cells persisting in the blood, spleen, lung, and tumors of NSG mice 7 days post-ACT (n=5). **(I)** Frequency of human $CD45^{+}$ cells in harvested tissues 5 days post-ACT with wtSiRPα-Fc and CV1-hIgG4-Fc T-cells (n=3). **(J)** Binding of soluble and immobilized recombinant SiRPα-Fc on A375 tumor cells (data representative of n=3 independent studies). Statistical analysis by one-way analysis of variance (ANOVA) (E-F and I) or unpaired, two-tailed t-test (H) with correction for multiple comparisons by post hoc Tukey's test (E-F and I). ****P< 0.0001; ***P < 0.001; **P < 0.01; *P < 0.05.

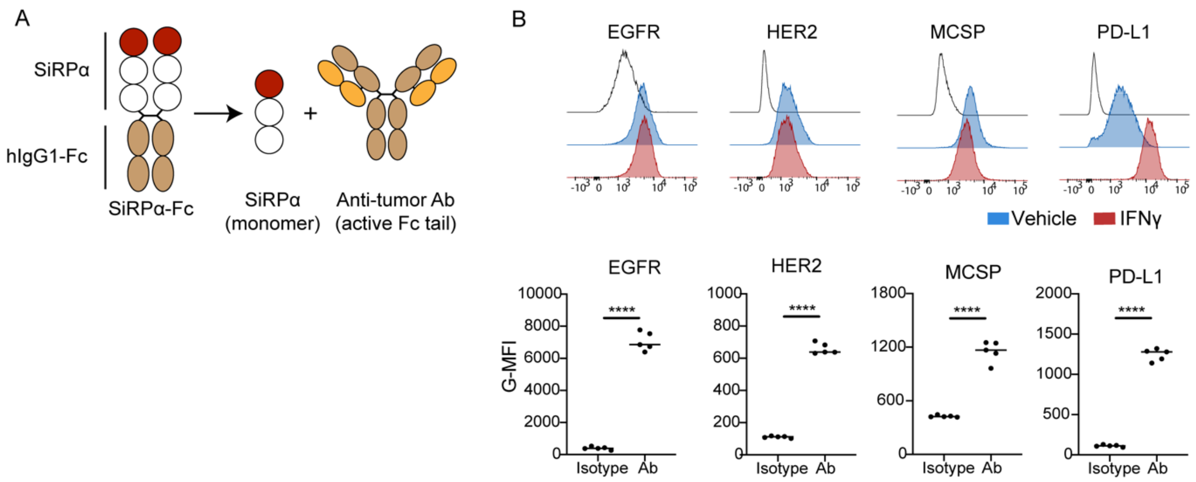

Supplemental Figure 6. Identification of suitable tumor-specific targets for monoclonal antibodies having antibody-dependent cellular phagocytosis (ADCP) activity. (A) Schematic of strategy to combine SiRPα-monomer with tumor-targeted Abs. **(B)** Expression of EGFR, HER2, MCSP, and PD-L1 on the surface of A375 tumor cells in vitro (top) and in established tumors in vivo (bottom) (n=5). Statistical analysis by unpaired, two-tailed t-test (B, bottom panel). ****P< 0.0001; ***P < 0.001; **P < 0.01; *P < 0.05.

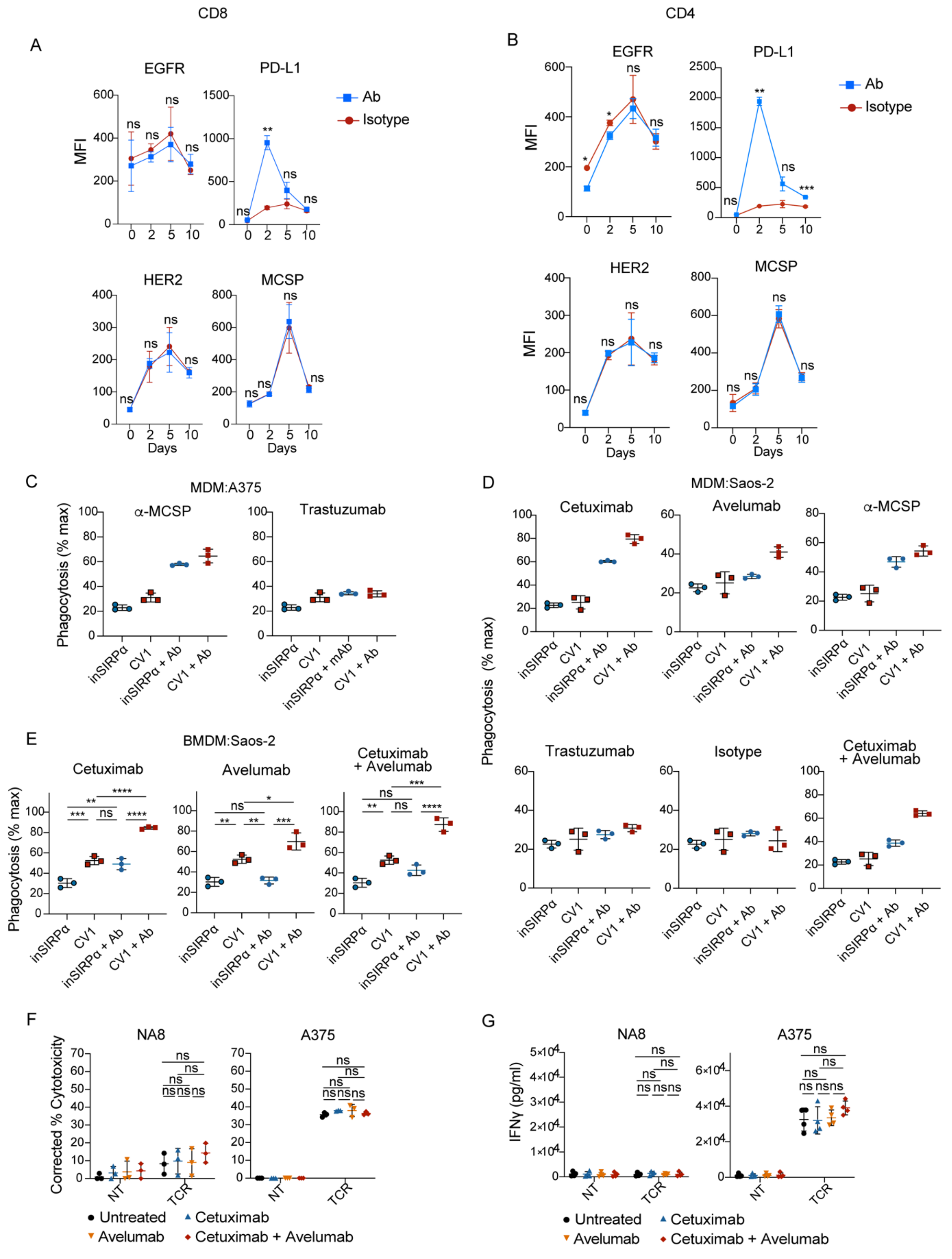

Supplemental Figure 7. **Tumor-targeted monoclonal antibodies cooperate with SiRPα-monomer secreted by gene-modified T-cells to augment macrophage-mediated phagocytosis of tumor cells. A)** Evaluation of EGFR, HER2, MCSP, and PD-L1 expression at different time points by cultured $CD8^{+}$T-cells (n=3) and by **(B)** cultured $CD4^{+}$ T-cells (n=3). **(C)** Human-MDM phagocytosis of A375

tumor cells in the presence of T-cell-secreted SiRPα-monomer along with Trastuzumab or anti-MCSP Abs (representative results for n≥3 donors). **(D)** Human-MDM phagocytosis of Saos-2 tumor cells in the presence of T-cell-secreted SiRPα monomer along with different tumor-targeted Abs (representative results for n≥3 donors). **(E)** Murine (NSG)-BMDM phagocytosis of Saos-2 tumor cells in the presence of T-cell-secreted SiRPα-monomer along with Cetuximab or/and Avelumab (n=3). **(F)** Frequency of AnnexinV$^+$DAPI$^+$ cells, corrected to tumor alone, 24h post-co-culture with A97L-T-cells at E:T=1:1 in the presence or not of Cetuximab or/and Avelumab (n=3). **(G)** T-cell-secreted IFNγ levels 24h post-stimulation with A375 tumor cells at E:T=1:1 in the presence or not of Cetuximab or/and Avelumab (n≥3). Statistical analysis by two-way analysis of variance (ANOVA) (A-B) or one-way ANOVA (E-G), with correction for multiple comparisons by post hoc Sidak's test (A-B) or post hoc Tukey's test (E-G). ****P< 0.0001; ***P < 0.001; **P < 0.01; *P < 0.05.

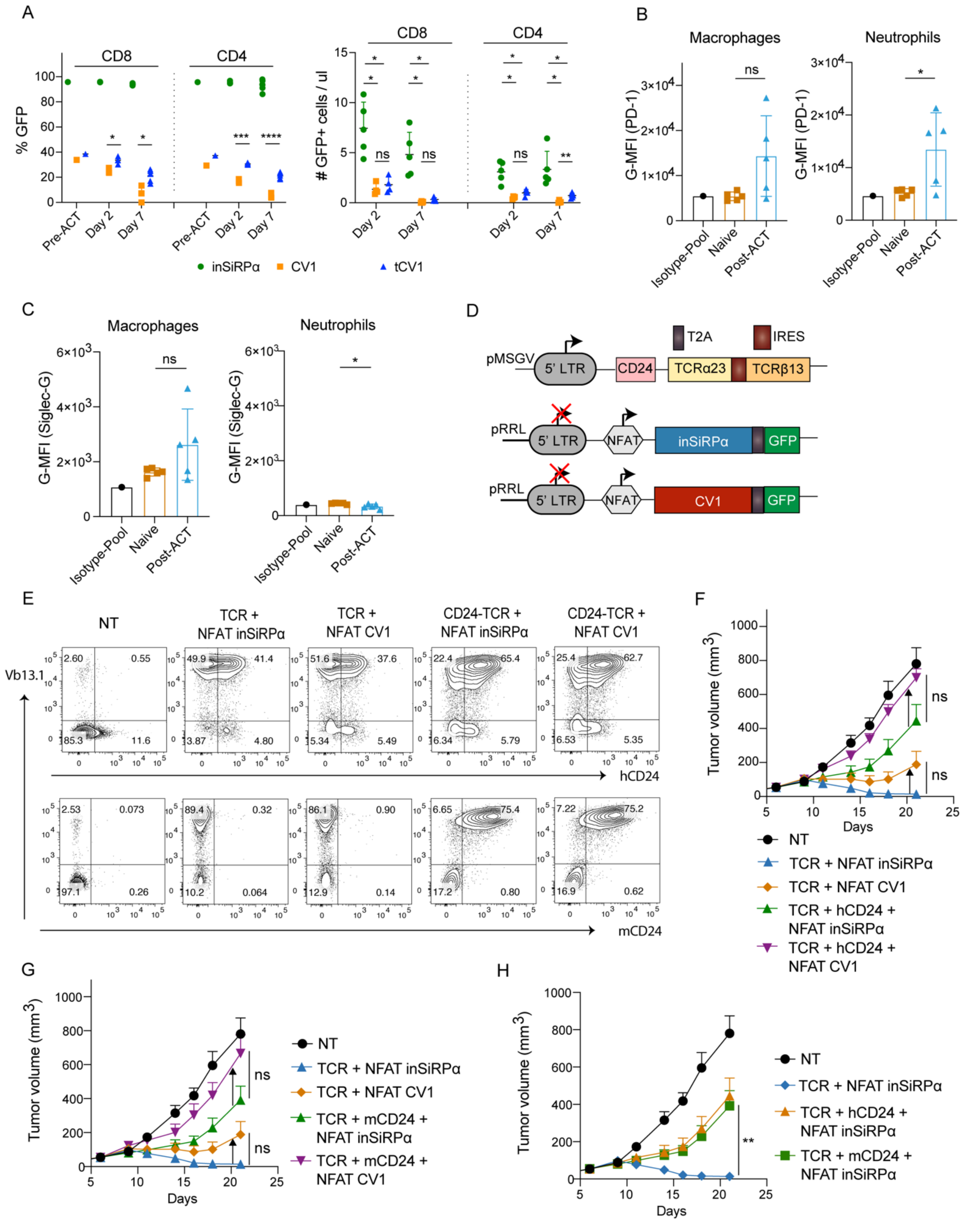

Supplemental Figure 8. SiRPα-monomer engineered human T-cells are phagocytosed by NSG macrophages but not human macrophages. (A) Frequency (left) and numbers per ul (right) of SiRPα-secreting GFP^{+} cells in the blood of NSG mice (n≥4, representative results from n=2 independent experiments). **(B)** Expression of mouse PD-1 on the surface of tumor-associated macrophages (left) and neutrophils (left) in established A375 tumors in vivo, pre- and post-ACT (n=5). **(C)** Expression of

mouse Siglec-G on the surface of tumor-associated macrophages (left) and neutrophils (left) in established A375 tumors in vivo, pre- and post-ACT (n=5). **(D)** Schematic of lentiviral constructs encoding SiRPα-decoys under 6xNFAT and of a retroviral construct encoding mouse or human CD24 together with A97L TCR. **(E)** Expression of human (top panel) or mouse (bottom panel) CD24 and A97L-TCR in transduced $CD8^+$ T-cells, detected by anti-CD24 and anti-Vβ13.1 Ab staining, respectively (data representative of n=3 donors). **(F-H)** A375 tumor growth and control curves following ACT with T-cells co-engineered with monomeric SiRPα decoys and human CD24 **(F&H)** or mouse **(G&H)** CD24 (n=8, representative results from n=2 independent experiments). Statistical analysis by two-way analysis of variance (ANOVA) (A and F-H) or unpaired, two-tailed t test (B and C) with correction for multiple comparisons by post hoc Tukey's test (A and F-H). ****$P < 0.0001$; ***$P < 0.001$; **$P < 0.01$; *$P < 0.05$.

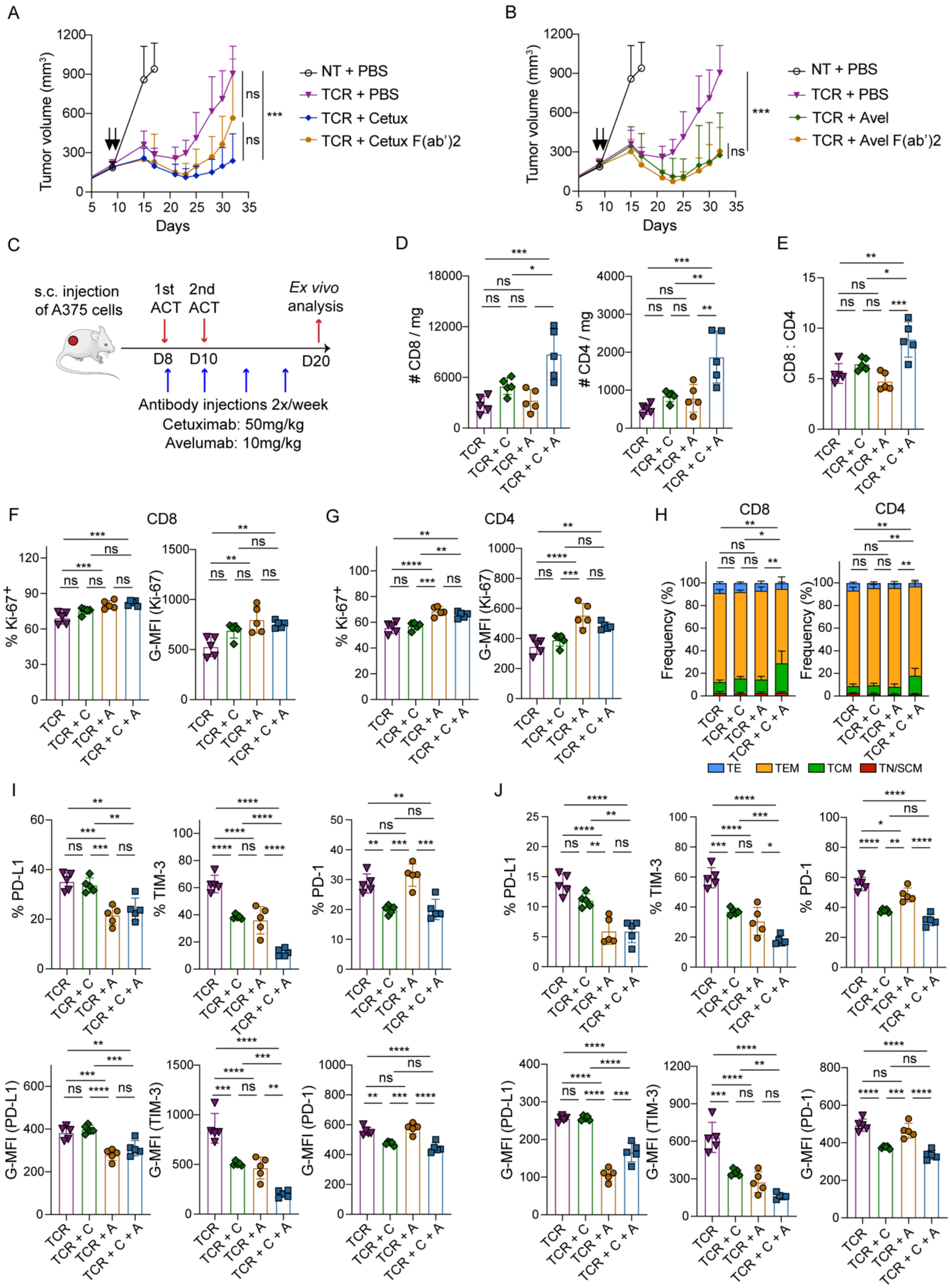

Supplemental Figure 9. Co-administration of tumor-targeted monoclonal antibodies augments the anti-tumor activity of adoptively transferred A97L-T-cells. (A) Control of A375 tumors in NSG mice following adoptive transfer of A97L-T-cells with co-administration of Cetuximab F(ab')2

fragments or Cetuximab ('C'; n=7). **(B) C**ontrol of A375 tumors in NSG mice following adoptive transfer of A97L-T-cells with co-administration of Avelumab F(ab')2 fragments or Avelumab ('A'; n=7). **(C)** Schematic of ACT study and ex vivo analysis 10 days post-ACT. **(D)** Number of intratumoral human $CD8^+$ (left) and $CD4^+$ (right) T-cells per mg of tumor (n=5). **(E)** Ratio of intratumoral $CD8^+$:$CD4^+$ human T-cell frequency (n=5). **(F)** Frequency (left) and G-MFI (right) of Ki-67 expression within intratumoral human $CD8^+$T-cells (n=5). **(G)** Frequency (left) and G-MFI (right) of Ki-67 expression within intratumoral human $CD4^+$T-cells (n=5). **(H)** Frequency of effector and memory phenotypes of intratumoral $CD8^+$ (left) and $CD4^+$ (right) T-cells (n=5) (Effector, T_E; Effector Memory, T_{EM}; Central Memory, T_{CM}; Naïve/Stem-cell like Memory, $T_{N/SCM}$). **(I)** Frequency (upper panel) and G-MFI (lower panel) of checkpoint receptors in $CD8^+$A97L-T-cells (n=5). (**J)** Frequency (upper panel) and G-MFI (lower panel) of checkpoint receptors in $CD4^+$A97L-T-cells (n=5). Statistical analysis by two-way analysis of variance (ANOVA) (A-B) or one-way ANOVA (D-J) with correction for multiple comparisons by post hoc Tukey's test (A-B&D-J). ****$P< 0.0001$; ***$P < 0.001$; **$P < 0.01$; *$P < 0.05$.

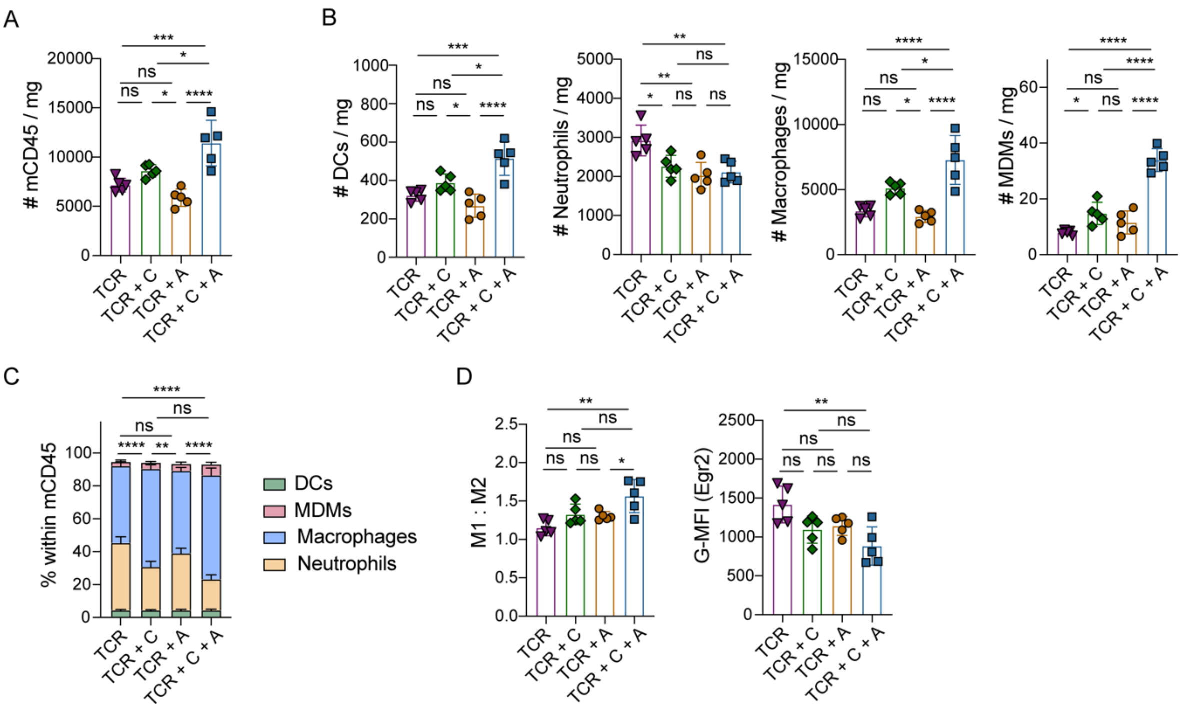

Supplemental Figure 10. Co-administration of tumor-targeted monoclonal antibodies mobilizes and activates the endogenous innate immune system. (A) Number of intratumoral mouse CD45$^+$ cells per mg of tumor 10 days post-ACT +/- Cetuximab ('C') or/and Avelumab ('A'; n=5). **(B)** Number of intratumoral mouse dendritic cells (DCs), neutrophils, macrophages, and monocytic-derived macrophages (from left to right) per mg of tumor (n=5). **(C)** Frequency of mouse myeloid populations within the CD45$^+$ compartment (n=5). (**D)** Ratio of M1:M2 macrophages based on the frequency of CD38$^+$ (M1) and Egr2$^+$ cells (M2) cells, and G-MFI of M2 marker Egr2 (right) (n=5). Statistical analysis by one-way analysis of variance (ANOVA) (A-D) with correction for multiple comparisons by post hoc Tukey's test (A-D). ****P< 0.0001; ***P < 0.001; **P < 0.01; *P < 0.05.

Supplemental Table 1. Antibodies used in flow cytometry experiments.

RESOURCE	Clone	Source	Identifier	Concentration
**Antibodies or stains for Flow Cytometry**				**µl per 100µl or working concentration**
Human CD4-Phycoerythrin (PE)	SK3	Biolegend	#344606	0.5ul
Human CD4-Allophycocyanin (APC)	SK3	Biolegend	#344614	1ul
Human CD4-Brilliant Violet 605 (BV605)	OKT4	Biolegend	#317438	0.5ul
Human CD4-Spark UV 387 (BUV387)	SK3	Biolegend	#344686	2ul
Human CD8-Fluorescein Isothiocyanate (FITC)	SK1	Biolegend	#344704	1ul
Human CD8- Allophycocyanin (APC)	SK1	Biolegend	#344722	1ul
Human CD8-Brilliant Violet 650 (BV650)	RPA-T8	Biolegend	#301042	0.5ul
Human/Mouse CD11b Phycoerythrin-Cy7 (PE-Cy7)	M1/70	Biolegend	#101215	1ul
Human CD45-Phycoerythrin-Cy5 (PE-Cy5)	H130	Biolegend	#304010	0.1ul
Human CD45-Brilliant Violet 785 (BV785)	HI30	Biolegend	#304048	0.5ul
Human CD45RA-ECD	2H4	Beckman Coulter	#B49193	0.5ul
Human CD47-Fluorescein Isothiocyanate (FITC)	CC2C6	Biolegend	#323106	1ul
Human CD47- Phycoerythrin-Cy7 (PE-Cy7)	CC2C6	Biolegend	#323114	1ul
Human CD64 (Fc receptor I)-Allophycocyanin	10.1	Biolegend	#305014	1ul
Human CD69-Alexa Fluor 700 (AF700)	FN50	Biolegend	#310922	1ul
Human CD137 (4-1BB)-Brilliant Violet 711 (BV711)	4B4-1	BD Biosciences	#740798	1ul
Human CD197 (CCR7)-Allophycocyanin-Cy7 (APC-Cy7)	G043H7	Biolegend	#353212	2ul
Human CD223 (LAG3)-PerCP-eFluor710	3DS223H	Thermo Fisher Scientific	#46-2239-42	1ul
Human CD274 (PD-L1)- Brilliant Violet 421 (BV421)	29E.2A3	Biolegend	#329714	1ul
Human CD279 (PD-1)-Allophycocyanin-Cy7 (APC-Cy7)	NAT105	Biolegend	#467415	1ul
Human CD340 (HER2)-Brilliant Violet 421 (BV421)	24D2	Biolegend	#324420	2ul
Human CD366 (TIM3)-Brilliant Violet 421 (BV421)	F38-2E2	Biolegend	#345013	1ul
Human EGFR-Phycoerythrin (PE)	AY13	Biolegend	#352903	2ul in cell pellet
Human MCSP (CSPG4)-Fluorescein Isothiocyanate (FITC)	EP-1	Miltenyi Biotec	#130-098-794	1ul
Human Ki-67-Brilliant Violet 421 (BV421)	Ki-67	Biolegend	#350505	2ul
Human TCR Vbeta13.1- Phycoerythrin (PE)	IMMU 222	Beckman Coulter	#IM2292	7ul in cell pellet
Human IgG Fc- Phycoerythrin (PE)	HP6017	Biolegend	#409304	0.5ul
Mouse CD11c- Brilliant Violet 605 (BV605)	N418	Biolegend	#117333	1ul

Mouse Egr2- Allophycocyanin (APC)	Erongr2	Invitrogen	#17-6691-82	2ul
Mouse CD38-Alexa Fluor 488 (AF488)	90	Biolegend	#102714	2ul
Mouse CD45-Phycoerythrin-Cy5 (PE-Cy5)	30F11	Thermo Fisher Scientific	#15-0451-82	0.2ul
Mouse CD45-Phycoerythrin-Cy5.5 (PE-Cy5.5)	30-F11	Thermo Fisher Scientific	# 35-0451-82	0.1ul
Mouse Ter119- Phycoerythrin-Cy7 (PE-Cy7)	Ter119	Thermo Fisher Scientific	# 25-5921-82	0.5ul
Mouse F4/80- Allophycocyanin-Cy7 (APC-Cy7)	BM8	Thermo Fisher Scientific	#47-4801-82	1ul
Mouse F4/80- Pacific Blue	BM8	Biolegend	#123124	2ul
Mouse Ly-6C-PerCP-Cyanine 5.5 (PerCP-Cy5.5)	HK1.4	Thermo Fisher Scientific	#45-5932-82	0.5ul
Mouse Ly-6G- Phycoerythrin-Cy7 (PE-Cy7)	1A8	Biolegend	#127618	1ul
Mouse CD172a (SiRPα)- Phycoerythrin-Dazzle (PE-Dazzle)	P84	Biolegend	#144015	1ul
Mouse IgG Fc-Phycoerythrin (PE)	Polyclonal	Antibodies-Online	#ABIN2669885	2ul
Annexin V-V500		BD Biosciences	#561501	2.5ul
Multimer against HLA-A*0201 / NY-ESO-1157-165 SLLMWITQC - Phycoerythrin (PE)		TCMetrix / Tetramer Core Facility in University of Lausanne		2ul in cell pellet
Multimer against HLA-A*0201 / Melan-A-MART-126-35 EAAGIGILTV - Phycoerythrin (PE)		TCMetrix / Tetramer Core Facility in University of Lausanne		1ul in cell pellet
Human TruStain FcX™ (Fc Receptor Blocking Solution)		Biolegend	#422301	10ug/ml
Purified Rat Anti-Mouse CD16/CD32 (Mouse Fc Block)	2.4G2	BD Pharmingen	#553142	10ug/ml
Invitrogen™ LIVE/DEAD™ Fixable Aqua Dead Cell Stain Kit, for 405 nm excitation		ThermoFisher Scientific	#L34957	1/200
DAPI (4',6-Diamidino-2-Phenylindole, Dilactate)		Sigma-Aldrich	#D9542	100ng/ml
